# Exploring the effects of methodological choices on the estimation and biological interpretation of life history parameters for harbour porpoises in Norway and beyond

**DOI:** 10.1371/journal.pone.0301427

**Published:** 2024-07-05

**Authors:** Anne Kirstine Frie, Ulf Lindström

**Affiliations:** 1 Institute of Marine Research, Tromsø, Norway; 2 UiT The Arctic University of Norway, Tromsø, Norway; Texas A&M University, UNITED STATES

## Abstract

This study investigates effects of subtle methodological choices on the estimation and biological interpretation of age, growth and reproductive parameters for harbour porpoises. The core analyses are based on a focal Norwegian data set built on samples from 134 harbour porpoises caught incidentally in gillnet fisheries along the Norwegian coast during autumn 2016 and spring 2017. Two contrasting practices for interpretation of seasonal and ontogenetic characteristics of tooth growth layer formation resulted in significant age differences among spring samples of young porpoises and for older animals across seasons. In turn, these differences affected estimates of age at maturity and asymptotic lengths, respectively. We also found significant differences in male age at maturity between two well-documented maturity criteria and between mathematical estimators of age at maturity for both sexes. Two different criteria for *corpus albicans* classification furthermore resulted in different patterns of ovarian *corpora* accumulation, which may affect some estimates of fecundity rates and contaminant loads. Both *corpora* accumulation patterns were also found in reanalysed data from German and Greenlandic porpoises. Based on tabulated overviews of methodological choices made in previous harbour porpoise studies, we argue that several of the issues mentioned above have wider relevance and may affect the validity of meta-analyses as a tool for estimating harbour porpoise sensitivity to extrinsic pressures. Differences in cause of death (COD) composition between data sets can have a similar effect. We demonstrate this in a meta-analysis of published harbour porpoise pregnancy rates, showing significantly higher values for trauma-killed samples compared to samples comprising mixed COD categories. COD also affected the estimated impacts of three previously analysed extrinsic predictors as well as an added predictor for vessel noise levels. We discuss the potential contributions of methodological, biological and anthropogenic factors in shaping observed regional differences in estimates of harbour porpoise life history parameters.

## Introduction

**Fig 1:**
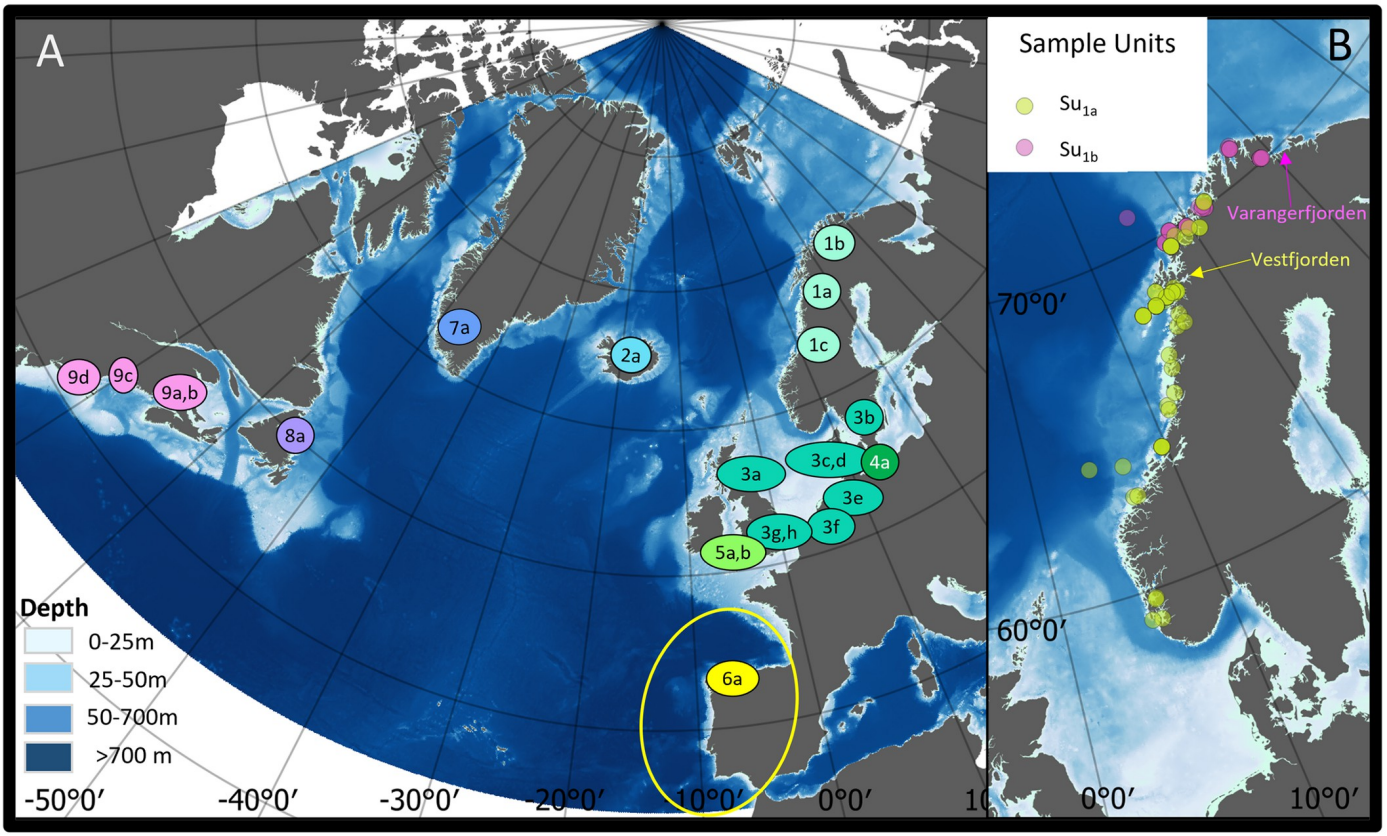
Distribution of data collection for North Atlantic studies of harbour porpoise life history parameters. (A) Geographic distribution of sample units (Sus) for the present study (Su_1a_ and Su_1b_) and previous studies of harbour porpoise life history parameters in the North Atlantic area. Number codes refer to geographic assessment units outlined by an expert workshop in 2018 [7]. Letters refer to different spatiotemporal units within geographic assessment units. More details on the underlying reference studies are given in the materials and methods section and in S1 Table. The colour scale of sample unit symbols qualitatively reflects relative genetic closeness identified by [2]. The yellow ring reflects the approximate distribution area of Iberian subspecies of harbour porpoises. (B) Bycatch locations of harbour porpoises in the focal Norwegian data set in autumn-winter 2016 (Su_1a_) and in winter-spring 2017 (Su_1b_). The map was produced in QGIS (qgis.org; version 3.26.2) using the Natural Earth data base (naturalearthdata.com).

Harbour porpoises (*Phocoena phocoena*) are widely distributed across the northern hemisphere but are particularly abundant in shelf waters of the North Atlantic region [1]. The subspecies *Phocoena phocoena phocoena* occupies the Atlantic region from Florida to Iceland and throughout Northwestern Europe. Studies have revealed significant isolation by distance in this area, particularly for maternally inherited markers [2] (see also Fig 1A), suggesting pronounced female philopatry. A distinct subspecies, tentatively named *Phocoena phocoena meridionalis*, has been identified along the Iberian and Northwest African coast [3–5], while harbour porpoises off West Greenland qualify as a distinct ecotype [6]. Furthermore, harbour porpoises in the Belt Sea and Baltic proper Sea are identified as two distinct populations [3].

Management units for harbour porpoises have been devised based on genetic, morphological, ecological or administrative criteria as outlined in [7, 8]. Of particular relevance to Norwegian waters and neighboring areas is the identification of significant genetic differences between harbour porpoises sampled on either side of 63°N [9]. Significant differences have also been found between harbour porpoises from Norway, Denmark, and the British North Sea based on nuclear genetic markers and scull measurements [10]. So far, no significant genetic differentiation has been found in studies based only on Norwegian samples [11]. Traditionally, however, Norwegian harbour porpoises to the North and South of 62°North, have been thought to constitute two different demographic units [12]. Further ecological differentiation of 3–4 geographic units along the Norwegian coast has been suggested based on differences in caesium levels [13]. In line with this information, various management bodies have generally recommended a precautionary subdivision of Norwegian harbour porpoises into 2–4 geographic assessment units [7, 8, 14]. Risk assessment models of incidental bycatches have, however, generally not taken potential differences between these assessment units fully into account [7, 14, 15], partly due to scarcity of area-specific life history data. Since existing compilations of harbour porpoise life history parameters show considerable variability between areas and studies [e.g. 16–19], the latter could be critical, particularly with respect to female reproductive rates used for population modelling [7, 14, 15, 20, 21]. Male reproductive parameters and somatic growth characteristics for both sexes, however, also show significant variability between studies [e.g. 16, 18, 19] and may inform assessments of population health and sensitivity to environmental changes and anthropogenic stressors.

Several studies have linked changes in harbour porpoise growth and reproductive rates to effects of anthropogenic pressures like contaminants [17, 22], anthropogenic noise [23, 24] and climate change [25, 26]. These extrinsic pressures do not only vary geographically but also in time, emphasizing the need for regular updates of life history data. Due to logistic and financial constraints on data collection, the spatiotemporal resolution of harbour porpoise life history data is, however, likely to always be suboptimal relative to the spatiotemporal variability of the many potential stressors [21, 22]. Therefore, it is important to consider “borrowing strength” from other studies and areas. So far this has generally taken the form of intuitive evaluations by experts in relevant fields (e.g. [20]), but more explicit quantitative analyses are emerging such as [17]. The validity of meta-studies, however, strongly depends on the comparability of the included estimates, which in turn depends on several methodological choices from selection of sample source to details of physical and mathematical analyses.

Significant steps have been taken to standardise the physical aspects of harbour porpoise life history studies through various international workshops resulting in protocols on morphological measurements and sampling of organs [27], determination of reproductive status [28, 29] and determination of age based on growth layer groups (GLGs) in tooth dentine [30–32]. Methodological variations are, however, still likely, even for age estimations explicitly performed according to [30–32]. The latter is partly due to the difficulties of discerning dentinal GLGs in animals > 7 years due to gradual closing of the pulp cavity [31–33]. These problems may give rise to variable degrees of underestimation as also reported for dentine-based age estimation of phocid seals [34, 35]. Blind-reading experiments based on known-age harp seal teeth have shown that only readers trained on known-age teeth have higher accuracy for older seals than less experienced readers [34]. In fact, the latter were generally less negatively biased than experienced readers without known-age training. Due to the near absence of known-age teeth for harbour porpoises, the absolute and relative accuracies of age readers for this species are presently unknown. This uncertainty may contribute to the large differences in maximum age seen between studies e.g. [17, 19, 36, 37]. Different opinions on inclusion of information from cementum layers [32, 38, 39] may also contribute to this pattern [35]. Another source of heterogeneity in age estimation arises from the choice of seasonal reference point for the assigned age. In harbour porpoises, the boundary layer completing a GLG is reported to form between late winter and summer [38, 39], while calving and breeding occurs between late spring and early autumn, depending on geographic location [17, 40]. Some individuals may therefore be assigned an integer age corresponding to the upcoming summer calving season. This may not matter much for analyses of continuous processes like somatic growth since overestimation of some ages may be compensated by underestimation of other ages. For analyses of discrete life history events like onset of maturity, however, the assigned age should always refer to the most recent relevant season, such as the most recent reproductive season. Measures to ensure this have, however, only been explicitly reported by one study [41].

The seasonal distribution of sampling also has implications for the criteria used to assess reproductive status of both males and females. Sperm production only occurs in the summer period [42, 43] and this seasonality affects several other traits used for assessment of male maturity status such as the diameter of seminiferous tubules [42–44] and combined testes weight [42]. Yet, few studies have explicitly considered seasonality in their choice of threshold values. For females, seasonal effects on pregnancy rates may be expected due to the cumulative incidence of abortions through the gestation period. Samples collected around the time of calving may furthermore be unreliable for estimation of female reproductive rates as some females may be sampled between reproductive cycles or just before their first ovulation [36, 37]. Using the total number of ovarian *corpora* as a relative indicator of reproductive activity reduces the sensitivity to seasonal effects and has sometimes been done for harbour porpoises [45, 46]. This, however, accentuates questions regarding the persistence and characteristics of various types of ovarian *corpora*, which do not appear to be settled for porpoises, or odontocetes in general.

Generally, harbour porpoises have been found to ovulate annually (e.g. [29]) and resulting *corpora lutea* (CLs) have been assumed to persist as a *corpus albicans* (CA) throughout life for all cetaceans [29, 47]. However, for some odontocetes, CAs from infertile ovulations appear to be less persistent than CAs from fertile breeding cycles [48, 49]. Even the latter seem to disappear within a few years in some species according to studies showing no increasing trend in CA numbers after the first years of reproductive life (e.g. [50]). A similar pattern has been found in harbour porpoises from the Bay of Fundy area [51], whereas later studies have reported a linear increase in *corpora* numbers and used this to infer reproductive parameters (e.g. [41, 47, 52]). Several studies have reported young or middle-aged harbour porpoise females with more *corpora* than can be attributed to annual ovulations within their estimated lifetime (45,47,51,53). This suggests that some females can ovulate more than once during the same season, and that the *corpora* from infertile ovulations are visible for some time [50, 53]. Other potential explanations could be underestimation of age or the presence of *corpora*-like structures arising from regression of follicles in various stages, so-called *corpora atretica* [29]. No clear species-specific description of these structures appears to exist [29, 51]. Some studies refer to descriptions made for fin whales (*Balaenoptera physalus*) [54], although descriptions made for other odontocetes like short-finned pilot whales (*Globicephala macrorhyncus*) [55] may seem equally, if not more appropriate. For the latter, *corpora atretica a*, are reported to be large, well-defined structures similar to CAs, but confined to the surface of the ovary [55]. Although most studies on harbour porpoise reproductive parameters claim to exclude *corpora atretica*, the differences in cited protocols suggest that heterogeneities in criteria for excluding *corpora atretica* could drive some of the observed differences in *corpora* accumulation patterns.

Mathematical estimators of age at maturity in harbour porpoises have generally been based on formulas averaging across unsmoothed age-specific proportions mature [19, 56, 57] or on fitted logistic curves [18, 19]. The former are sensitive to stochastic effects disturbing an overall asymptotic increase, while the latter are sensitive to any deviation from a symmetric sigmoid maturity curve. To our knowledge, no alternative parametric curves have been tested for proportions mature in harbour porpoises, but tests for other marine mammals have shown a better fit to asymmetric sigmoid curves [58]. For size-at-age analyses, both Gompertz and Von Bertalanffy curves have been used for harbour porpoises (e.g. [19, 41]).

A general concern for comparability of life history estimates between studies is biases due to different methods of specimen acquisition (sample source). Porpoises in chronically poor health are widely considered to be overrepresented in stranded samples, while less health-related biases are expected for specimens killed by sudden trauma such as gunshots or entanglement and asphyxiation in fishing gear (e.g. [17–19, 45]). Nevertheless, samples from different sources are often mixed and cause of death (COD) categories are not always taken explicitly into account in meta-studies (e.g. [17]).

All the methodological questions and choices listed above were considered during our efforts to obtain valid and comparable life history parameters from Norwegian harbour porpoises caught incidentally in bottom-set gillnets during autumn 2016 and spring 2017 (see distribution in Fig 1B). For this focal data set, the current study chose an exploratory approach to estimation of life history parameters based on alternative age readings, CA classification criteria, male maturity criteria, mathematical estimators and models. Acknowledging the general need for “borrowing strength” from other studies, we compiled overview tables with relevant methodological characteristics of published life history parameters for harbour porpoises. We also used the data from [17] and some additional sources to rerun a previously published meta-analysis of extrinsic effects on pregnancy rates. For this reanalysis, we added explanatory variables for cause of death (COD) and vessel noise (Noise) to the original set of explanatory variables for mean energetic density of diet (MEDD), PCB levels (PCB) and a cumulative human impact factor (CHI). Based on our findings, we evaluate the potential effects of methodological issues and extrinsic drivers on the understanding of regional differences in harbour porpoise life history parameters.

## Materials and methods

### Sampling and sample units of the focal Norwegian data set

The focal Norwegian data set comprised 134 harbour porpoises incidentally caught in gillnets along the Norwegian coast during autumn (September-October) 2016 (N = 73) and spring (February-April) 2017 (N = 61). The depth of the gillnets ranged from 20 to 120 m. The geographic hotspots of sampling (>50% of all samples) were Vestfjorden in 2016 and Varangerfjorden in 2017 (Fig 1B). The overall geographic range of sampling also differed between years. The samples from 2016 and 2017 thus represent a seasonal, an annual and a geographic split, which are accounted for in the analyses. The geographic sample split roughly mirrors the split between the northern and western-central units of four geographic areas chosen for ongoing modelling of regional sensitivities to bycatch rates in Norwegian waters [14]. In the following, samples from 2016 and 2017 are generally referred to as sample unit 1a (Su_1a_) and sample unit 1b (Su_1b_), respectively. Immediately after being landed, the porpoises were frozen whole. Gross post-mortem examinations and collection of teeth and reproductive organs were performed upon thawing according to [27].

Comparisons of life history parameters across existing studies will refer to the sample unit codes shown in Fig 1A. The spatiotemporal origin and reference study of the data for each sample unit are as follows: Su_1a_ and Su_1b_: Norway 2016 and 2017, respectively (present study); Su_1c_: Norway 1988–90 [36]; Su_2a_: Iceland 1991–97 [41]; Su_3a_: Scotland 1990–2005 [18]; Su_3b_: Western Sweden 1988–90 [36]; Su_3c_: Denmark 1985–91 [59]; Su_3d_: Denmark 1962–98 [60]; Su_3e_: German North Sea 1987–2016 [61]; Su_3f_: Dutch North Sea 2006–2019 [17]; Su_3g_: Southeastern UK 1990–99 [19]; Su_3h_: Southeastern UK 2000–2012 [19]; Su_4a_: German Baltic Sea [61]; Su_5a_: Southwestern UK 1990–99 [19]; Su_5b_: Southwestern UK 2000–2012 [19]; Su_6a_: Bay of Biscay 1990–2010 [62]; Su_7a_: West Greenland 1988–95 [52]; Su_8a_: Eastern Newfoundland 1990–91 [37]; Su_9a_: Bay of Fundy 1969–73 [63]; Su_9b_: Bay of Fundy 1985–89 [63]; Su_9c_: Gulf of Maine 1989–93 [47]; Su_9d_: Massachusetts 1975–89 [64]. The number part of the sample unit codes refers to separate assessment units outlined by an expert workshop in 2018 [7] (see also overview with author-year style citations in S1 Table). Letters of the sample unit codes refer to different spatiotemporal units within geographic assessment units.

The study areas used in the existing literature did not align fully with the geographic assessment units recommended by [7]. This is for example true for the Irish and Celtic Seas (i.e. Southwestern UK) in [19], Eastern and Western Scotland in [18], Danish North and Belt Seas in [59, 60] and the Norwegian North Sea and more northern assessment unit along the Norwegian coast in [36]. Since it is not possible for us to change the spatial organisation of published analyses to fit the assessment units delineated in [7], we have assigned the data for the Irish and Celtic Sea presented in [19] to a pooled unit, data from both sides of Scotland in [18] to the North Sea, all Danish data from [59, 60] to the North Sea and Norwegian data from [36] to a pooled unit for the Norwegian and Barents Seas. The same was done for the focal Norwegian data set of the present study due to the very low incidence of specimens from the North Sea (see Fig 1B). The age at maturity data for the German North Sea and the German Baltic Sea were pooled in the original study [61] and are presented for both sample units in the present study.

### Age estimation

Two teeth from each animal were selected for age determination and decalcified and stained according to [31, 32]. One tooth was sectioned at right angles to the jaw (the dolphin-cut) and the other was sectioned in parallel to the mandible/gum line (the porpoise cut) following [32]. The teeth were sectioned through the pulp cavity, to ensure visibility of all GLGs. In harbour porpoises, a dentinal GLG viewed under transmitted light usually consists of a thicker, opaque layer deposited over the main part of the year and a thinner, translucent layer deposited towards the end of the annual breeding cycle [32]. This thin layer is often referred to as the boundary layer [65], as it marks the completion of a GLG. The other layer will be referred to as the main growth layer in the present study.

The total set of dolphin and porpoise cut sections was first read by a reader (R1) with extensive experience in age estimation of porpoise teeth (>1000 teeth). R1 provided estimates of integer age based on the number of complete GLGs (GLG age). Sometimes notes were made of partial GLGs, which were expressed as the decimal proportion of the width of the developing GLG at the time of death compared to the previous complete GLG. The number of complete GLGs plus any additional partial GLG constitutes the decimal GLG age (GLG+). Only the ages based on complete GLGs by R1 (R1_GLG_) were, however, used for further analyses, as these were most consistently recorded and reflect the core definition of GLG-based ages in existing guidelines [31, 32]. The second reader (R2) was primarily trained in age estimation of harp seals (*Pagophilus groenlandicus*) and grey seals (*Halichoerus grypus*) based on dentine and cementum, respectively. The training included participation in the known-age reading experiments for harp seals [34] (reader N1’) and grey seals [66] (reader Nb1) as well as basic training in age estimation of harbour porpoises. R2 explicitly aimed to estimate the integer age at the animals most recent birthday to ensure a common seasonal reference point for all individuals regardless of sampling season. These ages are referred to as standard ages by R2 (R2_Stdg_). The accuracy of R2_Stdg_ depends on the readers ability to determine whether the last full GLG in spring samples was completed in the current or previous year cycle. This assessment was based on visual standards established during reading of autumn samples for the age class-specific thickness of the developing main growth layer relative to the main growth layer of the previous age class. The season of sampling was known to both R1 and R2, and both readers read autumn samples before spring samples.

Both readers made separate age estimates for porpoise and dolphin cut sections. For R1, these estimates were primarily based on dentine, although cementum was sometimes taken into consideration. R2 made explicit separate estimates for cementum layers and for the top and root sections of the dentine for both porpoise and dolphin cut sections. Both readers arrived at their final age estimate through a holistic evaluation of all readings, based on subjective weightings of the quality of each section/tissue/reading position rather than simple averaging. This is in line with the standard protocol [32]. Since the general method of sample preparation and understanding of GLG structure is the same for R1 and R2, we refer to the two sets of readings as age reading practices (sometimes abbreviated AgeRp’s in the present study). Differences in seasonal reference point for age estimates are referred to as seasonal age assignment criteria (sometimes abbreviated sAgeCrit in the present study). In addition to the already mentioned sAgeCrits (GLG, GLG+ and Stdg), we also refer to decimal age (Decg), rounded GLG age (GLG++) and GLG’ age (seasonal correction only for individuals<1 year of age). Decg is calculated by adding a seasonal correction term to Stdg. This term is the decimal fraction of a year passed between the estimated sample peak date of birth (see next section) and the animals date of death. Since Decg has only been explicitly estimated by R2 in the present study, these ages are referred to as R2_Decg_. GLG_++_ is GLG+ rounded to the closest integer age. An intermediate version of ages rounded to the nearest half GLG is sometimes used (for example by [41]) but is referred to as GLG+ in the present study. Seasonal correction only for zero-year-olds (GLG’ ages) has been applied by [19] based on rounding to the nearest quarter year based on the relative thickness of the developing main growth layer. The present study estimated a variant of GLG’ for the focal Norwegian data set by substituting estimates of R1_GLG_ = 0 with the seasonal correction term estimated for the same animals by R2. This resulted in a fourth age reading practice termed R1_GLG’_. Effects of all four mentioned age reading practices on modelled length-at-age parameters were explored for the focal Norwegian data set.

### Foetal growth and peak dates of conception and birth

The date of conception of pregnant females (N = 22) was back-calculated from the foetal size [40, 67]. The foetal age, defined as *t* (in days), is defined as:

$$t=\left(L_{t}/u\right)*30.5+t_{0}$$ (1)

Where *L*_*t*_ is the foetal length at age *t (mm)*, *u* is the foetal growth rate (mm/month), 30.5 is the average number of days in a month, and *t*_*0*_ is the estimated duration of the lag phase in days. The lag phase of placental mammals can be estimated as:

$$t_{0}=7.25*{m_{neo}}^{0.19}$$ (2)

Where *m*_*neo*_ is the birth weights of the porpoises in grams. The growth rate (*u*) was estimated by regressing foetus length on the month of death (starting at 1 January 2016 and running to 24 December 2017). Since there are no data on birth weights of Norwegian harbour porpoises, we used the estimated average value from nearby areas in the Kattegat, Skagerrak and North Sea of 6025 ± 440 g [40]. Based on this, *t*_*0*_ was estimated at 37.9 days. The peak date of conception was calculated by subtracting the foetus age (days) from the catch date. The gestation length was estimated by substituting *L*_*t*_ in Eq (1) with the mean length at birth (762 ± 54 mm; [40]). An approximate standard date of birth was calculated by adding the number of days of the lag phase and the gestation time to the estimated peak date of conception.

### Postnatal growth

Effects of age reading practice, sex and sample unit on length-at-age was initially explored in general additive models (GAMs) with normal errors using the ‘*mgcv*’ package [68] for the statistical program, R, version 3.6.1, [69]. Von Bertalanffy and Gompertz three-parameter curves for length were also fitted to the length-at-age data using the parameterisation presented in [70]. AIC for these two curve fits were virtually identical across sexes, readers and seasons (ΔAIC<1) and only the Gompertz curve shown in (3) was therefore used in subsequent analyses.:

$$L_{t}\;=L_{\infty }{\left(\frac{l_{0}}{L_{\infty }}\right)}^{\text{exp}[\frac{k_{0}t}{l_{0}\;\text{ln}\left(\frac{l_{0}}{L_{\infty }}\right)}]}$$ (3)

Where *L*_*t*_ is the body length (cm) at age *t*, *L*_*∞*_ is the asymptotic body length in cm, *l*_*0*_ is the body length at age zero and *k*_*0*_ is the growth rate (cm/year) at age zero. The models were fitted separately for each sex in Excel^®^2016 using SOLVER to maximize the likelihood values for a normal distribution. The significance of differences in Gompertz parameters between sample units and age reading practices were assessed by comparisons of Akaikes Information criterion (AIC) [71] for models with different configurations of constraints on the Gompertz parameters. The completely unconstrained model had three independent Gompertz parameters for each combination of sample unit and age reading (i.e. four different data subsets). The overall significance of differences across the two sample units and the two age reading practices was tested by comparing the AIC of the unconstrained model with a model with identical Gompertz parameters for all four data subsets. If this difference is significant (ΔAIC>2), it is determined whether a common Gompertz model can be fitted within readings or within sample units without increasing AIC by more than 2 units. The most parsimonious of these options is the main result, but further constraints are tested to achieve the most parsimonious model. To estimate confidence intervals for the parameters, the final model was refitted with the nonlinear least squares function in the ‘*nlstools*’ package in the statistical program, R, version 3.4.3 [72].

### Reproductive status and parameters of females and males

Complete ovarian data were available for a total of 50 females. The ovaries were examined for the presence of *corpora lutea* (CLs) and *corpora albicantia* (CAs) by slicing them in 2 mm thick sections. The initial identification of CAs was based on guidance by a highly experienced reader of harbour porpoise ovaries, who was also responsible for ovary analyses in a West Greenland study [52], later included in a comparative analysis. For the focal Norwegian data set, separate records were kept of CAs which were visible on the surface but did not extend deeper into the interior of the ovary than they extended above the plane of the ovary surface. These are referred to as surface CAs, while other CAs are referred to as interior CAs and unspecified CA counts are referred to as total CAs. The *corpora* numbers resulting from adding the CL to the estimated numbers of interior or total CAs are referred to as interior *corpora* counts and total *corpora* counts, respectively. A female with a CL and/or a CA (both types) and/or a foetus (in case of incomplete ovary records) was considered sexually mature. For samples collected in 2017, the overall appearance of the uterus was characterised as mature or immature based on size, thickness of the uterine walls and the presence/appearance of stretch marks [28]. These characteristics were, however, not used in the primary classification of maturity status. The same is true for information on lactation status. Due to an unfortunate mistake, presence of milk in the mammary glands was only examined for the nine pregnant females bycaught in 2017. For these females, both mammary glands were cut open with a scalpel and examined macroscopically for exuding milk (dr. med. vet Katrine Ryeng, pers. comm.). Adult pregnancy rates were estimated as the proportion of sexually mature females, carrying a foetus at the time of death.

Among the eight females with incomplete ovarian data, five females with R2_Stdg_ of 3–11 years had a foetus and were included in calculations of age at maturity. Three females with R2_Stdg_ of zero, one and 18 years had neither a foetus nor valid ovarian data and were excluded from calculations of age at maturity. This was not considered likely to affect the calculations of age at maturity, as none of the excluded females were in the indeterminate age classes with respect to sexual maturity. Mean age at maturity (MAM) was estimated according to [58] based on Richards maturity curves [73] using the parameterization of [74]:

$$\hat{P}\left(x\right)=\hat{P}\infty {\left\{1-\left[1-m\right]\;\text{exp}\;\left[-\frac{k\left(x-M\right)}{m^{\frac{m}{1-m}}}\right]\right\}}^{1/\left(1-m\right)}$$ (4)

where $\hat{P}\left(x\right)=$ estimated proportion mature at age x (years), $\hat{P}\infty =$ asymptotic value set to 1, M = age at the point of inflection (years), k = slope at the point of inflection and m = shape parameter. MAM is determined as:

$$MAM=\text{w}+1-\sum_{x=1}^{x=w}\hat{P}\left(\text{x}\right)$$ (5)

where $\hat{P}\left(x\right)=$ estimated proportion mature at age x (years) and w = oldest age group in the sample (years). If $\hat{P}\left(w\right)=1$, this expression is equivalent to the more well-known formula presented in [57] formula. If $\hat{P}\left(w\right)<1$, Eq (5) assumes that all animals will be mature at age w+1. The same estimates will be obtained by applying the “sum of fractions immature” (SOFI) method outlined by [56] to exact mirror images of the data used in Eq (5). This method tends to be used for unsmoothed age-specific maturity data [18, 19, 36, 41]. For comparability with these studies, we estimated age at sexual maturity based on SOFI (ASM_SOFI_) for unsmoothed proportions immature in the present sample and estimated MAM based on Richards maturity curves for published data from other studies.

A third commonly used estimator of age at maturity is the age when 50% of the females are estimated to be mature (A_50_). This estimator was determined from the parameters of the Richards maturity curve as:

$${\text{A}}_{50}=M-\text{ln}\left(\frac{1-0.5^{\left(1-m\right)}}{1-m}\right)*{\frac{m}{k}}^{\left(m*\left(1-m\right)\right)}$$ (6)

The Richards models were fitted in Excel^®^2016, using SOLVER to maximize the log likelihood values for a binomial distribution (see also [58]). Support intervals approximating 95% confidence intervals were calculated for all parameter estimates according to [58]. Effects of sample units and age reading approach were tested by imposing constraints and comparing AIC values as explained for the Gompertz length-at-age models. Richards models can be forced to take the shape of more commonly used growth models like the logistic, Gompertz and von Bertalanffy curves by imposing constraints on the shape parameter [58–73]. This relationship was used to test the effects of estimating A_50_ based on the logistic curve as in most previous studies [17–19] compared to using Richards curves with an unconstrained shape parameter.

Patterns of age-specific accumulation of *corpora* were analysed with GAMs. For comparability with previous studies, only females with at least one ovarian *corpus* were included. Effects of different age reading, and CA classification practices were tested for the focal Norwegian data set. Published data for Germany [61] and West Greenland [52] were later included in comparisons of age-specific *corpora* accumulation patterns using GAMs with normal errors. These two data sets differed with respect to cited CA classification protocol, as [52] refers to [54], while [61] cites a less detailed generic protocol for odontocetes [29] and also uses a histological staining procedure to guide CA identification.

For the focal Norwegian data set, back-calculated ages at maturity were obtained for 19 mature females with complete ovary records by subtracting the number of CAs from R1_GLG_ and R2_Stdg_, respectively. Back-calculated ages at maturity <2 years were deemed unrealistically low. This threshold value was based on the lowest age estimate for a female with an ovarian *corpus* in the focal Norwegian data set. The same value was found by [37], while other studies have reported values from one year [36, 41] to three years [47, 52, 61]. Ovarian *corpora* that cannot be accounted for by annual ovulations since the second year of age, are termed “excess *corpora*” in the present study. Back- calculated ages at maturity>4 years were considered unrealistically high for the focal Norwegian data set because four years was the highest age assigned to a female with full ovary record and no sign of maturity. Other studies have reported a maximum female immature age of three years [37] or 5–14 years [61]. Factors affecting the probability of unrealistic back-calculated ages at maturity were analysed with binomial GAMs using a logit link function. Separate analyses were conducted for unrealistically low and high back-calculated ages at maturity.

Testes were collected during autumn 2016 (N = 41) and spring 2017 (N = 34) and stored in 10% buffered formalin. After fixation, the epididymis was removed, and the testes were weighed individually. No histological analyses were used to determine the maturity status of males in the Norwegian focal data set. A combined testis weight (CTW) of 200g has been recommended as a macroscopic maturity criterion for male harbour porpoises [75]. Previous comparisons of CTW and histological signs of maturity in males sampled well out of the breeding season, however, seem more in line with a threshold CTW for maturity at 100g [41, 76]. We therefore calculated estimators of age at maturity for criteria based on both CTW>200g and CTW>100g. These two criteria are sometimes abbreviated CTW_200g_ and CTW_100g_ in the present study. Mathematical estimators and estimation methods for male age at maturity were the same as for females.

### Meta-analyses of extrinsic and intrinsic effects on pregnancy rates

For our extended meta-analysis of pregnancy data formerly analysed by [17] (Meta-Analysis 1), we extracted the previously used 15 data points from the supplementary data Table 3 of [17]. All these data points had a complete set of information on pregnancy rate and the predictor variables MEDD, PCB1 (hereafter referred to as PCB) and CHI. Thirteen data points were derived from studies already assigned to eight of the nine assessment units shown in Fig 1 of the present study. The data set used by [17] furthermore comprised a data point for the Kattegat and Skagerrak Seas between Sweden and Denmark based on an unpublished study [77]. This data point was assigned to the already defined assessment unit 3b. It should, however, be noted that life history parameters for sample unit 3b elsewhere in the text are derived from [36]. A new sample unit 10a was assigned to a data set from the Salish Sea in the Northeast Pacific [78], which is not shown in Fig 1. Estimation of pregnancy rates for [78] differs from all the other studies by relying on a purely size-based criterion for maturity status. No estimates of pregnancy rate have been published for West Greenland harbour porpoises and sample unit 7a is therefore not included in the meta-analysis.

Of the included 15 data points [17], claimed that pregnancy rates were foetus-based in 12 cases and *corpora*-based in three cases, namely for 3b [77], 3e [61] and 4a [61]. After reading the underlying studies, we found that also the data points for Eastern Newfoundland (Su_8a_] and the Bay of Fundy (Su_9a_ and Su_9b_), were at least partly based on the presence of CLs according to [37, 63], respectively. The former found no foetuses at all and also suspected that sampling was done too early in the summer to include all ovulations. The number of ovulated and mature females shown for [37] in [17] appears to be based on inclusion of one female with a large follicle among the pregnant. The sample sizes for mature and ovulated females given for the German North Sea (Su_3e_) and Baltic Sea (Su_4a_) samples in [17] appear to be sourced from the supplementary data file to the referenced study [61]. The maturity criterion is, however, not quite clear as 14 females without *corpora* will have to be included to match the number of mature females given in [17]. These could possibly have been classified as mature based on age, length or the presence of follicles given in the supplementary data file, but no criteria are mentioned.

The assignment unit number of the sample unit name was used as a random effect for area in mixed effects modelling of extrinsic and intrinsic variables potentially affecting harbour porpoise pregnancy rates (see formula (7)). An overview of the data sets with associated area codes and other meta data is given in our S2 Table. A minor correction [+0.03] was made to the proportions pregnant given for the recent Norwegian sample, compared to [17]. The latter was based on [7], which was later found to rely on incomplete foetus data and preliminary CA counts.

In addition to the extrinsic variables explored by [17], we also tested the effects of annual average levels of large vessel noise on porpoise pregnancy rates. These data were extracted from a global map in Fig 2a of [79] and are measured in dB re 1uPa^2^ at 100 Hz. Maximum and minimum vessel noise levels for each study area were extracted by eye from an image enlarged to pixel size based on the given colour code. The delineation of areas included in the estimation of vessel noise levels was based on maps of sampling areas for each study of pregnancy rates, but also included the adjacent shelf areas. For the Danish data point, shelf areas all around Denmark were included. This extraction of vessel noise exposure data is approximate but given the overall uncertainties of the annual distribution pattern of the females sampled for reproductive data, we believe this level of accuracy is acceptable. The extracted vessel noise levels for each sample unit are shown in our S3 Table together with the values of the other predictor variables for each data point. The hearing range of harbour porpoises is generally estimated above 100 Hz, but behavioural reactions to noise from large vessels have nevertheless been documented and are of concern, since this is the main source of noise in most harbour porpoise habitats [80]. Because the available data set both comprises purely trauma-killed samples (mainly bycaught or hunted samples) and samples with mixed causes of death (stranded samples), we included a cause of death factor (COD) as a fifth predictor in the model. This was a binary categorical predictor with one level for pure trauma-killed samples and another level for samples with mixed causes of death.

Because trauma-killed samples are generally expected to be most representative for the average population, we also ran a separate analysis based only on this COD category (Meta-Analysis 2). For this analysis we added two data points comprised by trauma-killed sample subsets from Dutch waters and UK waters, derived from [17, 22], respectively (see also S2 Table). The latter sample is most likely a subset of the mixed COD samples presented in [18, 19].

A generalized linear random mixed model (GLMM), with a binomial distribution and logit link function, was used to explore the effect of extrinsic variables on pregnancy rates:

$$Logit\;\left(p\right)=\beta_{0}+\beta_{1}COD+\beta_{2}Noise+\beta_{3}MEDD\;+\beta_{4}CHI\;+\beta_{5}PCB\;+\;Area+\varepsilon$$ (7)

Where *p* is the probability of being pregnant, *β*′s are parameters to be estimated, Area is a random effect (intercept) and *ε* is the error term, the latter two were assumed to be normally distributed around zero. Interaction effects were only tested for models with two predictors. The marginal and conditional coefficients of determination (R^2^m and R^2^c) were calculated using the MuMIn package in R [81]. The GLMMs were fitted using the lme4 package [82] in R [69]. Models were selected based on comparisons of AIC. To be significant a predictor must reduce AIC of the model by more than two units [83]. Pearson product moment correlations were tested for all pairs of explanatory variables for the total data set and separately for the two COD categories using the generic function in R [69].

### General notes on statistical models and reporting of results

Statistical analyses not specifically mentioned above, were conducted in the ‘*mgcv*’ package, version 1.8–40 [68], for the statistical program, R, (version 3.6.1, [69]). This package was used both for GAMs (with at least one smooth parameter) and GLMs (for linearized continuous explanatory variables and/or factor variables). Binomial response variables were tested in models with binomial errors and logit links. Continuous response variables were analysed in models with identity link and normally distributed errors. The chosen estimation method was maximum likelihood (method =“ML”) for all types of models. Differences in AIC between specified models are generally abbreviated ΔAIC. Differences between a mentioned model and an intercept-only model is given as ΔAIC_itcpt_. Intervals given with a ± in parentheses are 95% confidence intervals unless otherwise stated.

## Results

### Differences between age reading practices

For the focal Norwegian data set, effects of sex, sample unit and age reading practice on mean age were tested for each of the postnatal stages in Table 1 using GAMs with normally distributed errors.

**Table 1:** Basic parameters by sex, life history stage and sample unit of harbour porpoises sampled in Norwegian coastal waters in 2016 (Su_1a_) and 2017 (Su_1b_).

Sample	N	Length (SD)	Mass (SD)	R1_GLG_	R2_Stdg_
				Range, Mean (SD)	Range, Mean (SD)
**Females Total**	**58**				
Mature Su_1a_	14	156.5 (6.7)	57.2 (9.0)	3–7;4.4 (1.3)	3–18; 6.8 (4.2)*
Mature Su_1b_	11	158.5 (8.2)	60.5 (7.1)	3–7; 5.4 (1.4)	2–22; 8.1 (5.7)*
Immature Su_1a_	13	133.5 (7.2)	35.9 (5.8)	1–3; 1.4 (0.7)	1–3; 1.5 (0.7)
Immature Su_1b_	12	140.5 (9.6)	43.1 (7.0)	1–4; 2.5 (0.8)	1–3; 1.0 (0.9)
Calves Su_1a_	4	108.3 (3.2)	22.8 (4.4)	0–0; 0.0 (0.0)	0–0;0.0 (0.0)
Calves Su_1b_	4	125.0 (5.4)	34.5 (3.3)	0–1; 0.75 (0.5)	0–0;0.0 (0.0)
Foetuses Su_1_; Su_1b_	2;7				
**Males Total**	**76**				
Mature Su_1a_	19	145.0 (6.1)	44.7 (4.2)	2–6; 4.0 (1.3)	2–16; 4.8 (3.2)
Mature Su_1b_	25	146.6 (6.5)	48.1 (6.5)	2–12; 6.5 (3.0)	2–16; 7.5 (4.4)
Immature Su_1a_	10	128.5 (9.2)	33.1 (4.8)	1–3; 1.6 (1.0)	1–3; 1.9 (0.9)
Immature Su_1b_	7	130.3 (5.5)	35.5 (3.2)	1–3; 1.4 (0.5)	1–3; 1.0 (0.0)
Calves Su_1a_	13	112.3 (7.9)	25.8 (4.0)	0–0; 0.0 (0.0)	0–0; 0.0 (0.0)
Calves Su_1b_	2	119.0 (5.7)	30.8 (0.4)	0–1; 1.0 (0.0)	0–0; 0.0 (0.0)
Foetuses Su_1a_; Su_1b_	7;2				

N: Number of samples; Length: Horizontally measured total length in cm; Mass: Total weight of carcass in Kg; R1_GLG_: Age in years estimated by reader 1 as number of completed GLGs; R2_Stdg_: Age in years during the most recent reproductive season estimated by reader 2; SD: Standard deviation of parameters; Note: Within the first year of age, individuals are assigned to the calf stage. Males with a combined testes weight of 100 grams are considered mature, one male caught in 2016 and estimated to be two years old (both R1_GLG_ and R2_Stdg_) had missing testes data but was included as immature in this table; Females with a foetus or a CA were considered mature. Three females aged zero, one and 7–18 years in 2016 but with missing reproductive data were classified as calf, immature and mature in this table.* denotes a significant difference between readings (*p*<0.05, ΔAIC<-2).

Significant effects were only found for females in the mature stage, which showed significantly higher (*p*<0.05) mean age for R2_Stdg_ (by 1.5± 1.1 years) and Su_1b_ (by 2.0 ± 1.1 years) (Δ_icpt_ = -14.5). Some of the individual differences between age estimates were quite remarkable, most notably for a female aged four years by R1_GLG_ and 22 years by R2_Stdg_. Photos of this specimen in our S1 Fig show a clear pattern of four GLGs in the dentine (S1A Fig), but a very thick layer of cementum (S1A and S1B Fig) by comparison with another female estimated at four years by both age reading practices (S1C Fig). Reader 2 counted 22 GLGs in the cementum of the first female, which contributed strongly to the final R2_Stdg_ estimate. For this female, the cementum layer appears to have closed the root opening to the pulp cavity at an early age (see S1A Fig), which may have stopped the blood supply to the dentinoblasts and thereby the formation of dentine.

**Fig 2:**
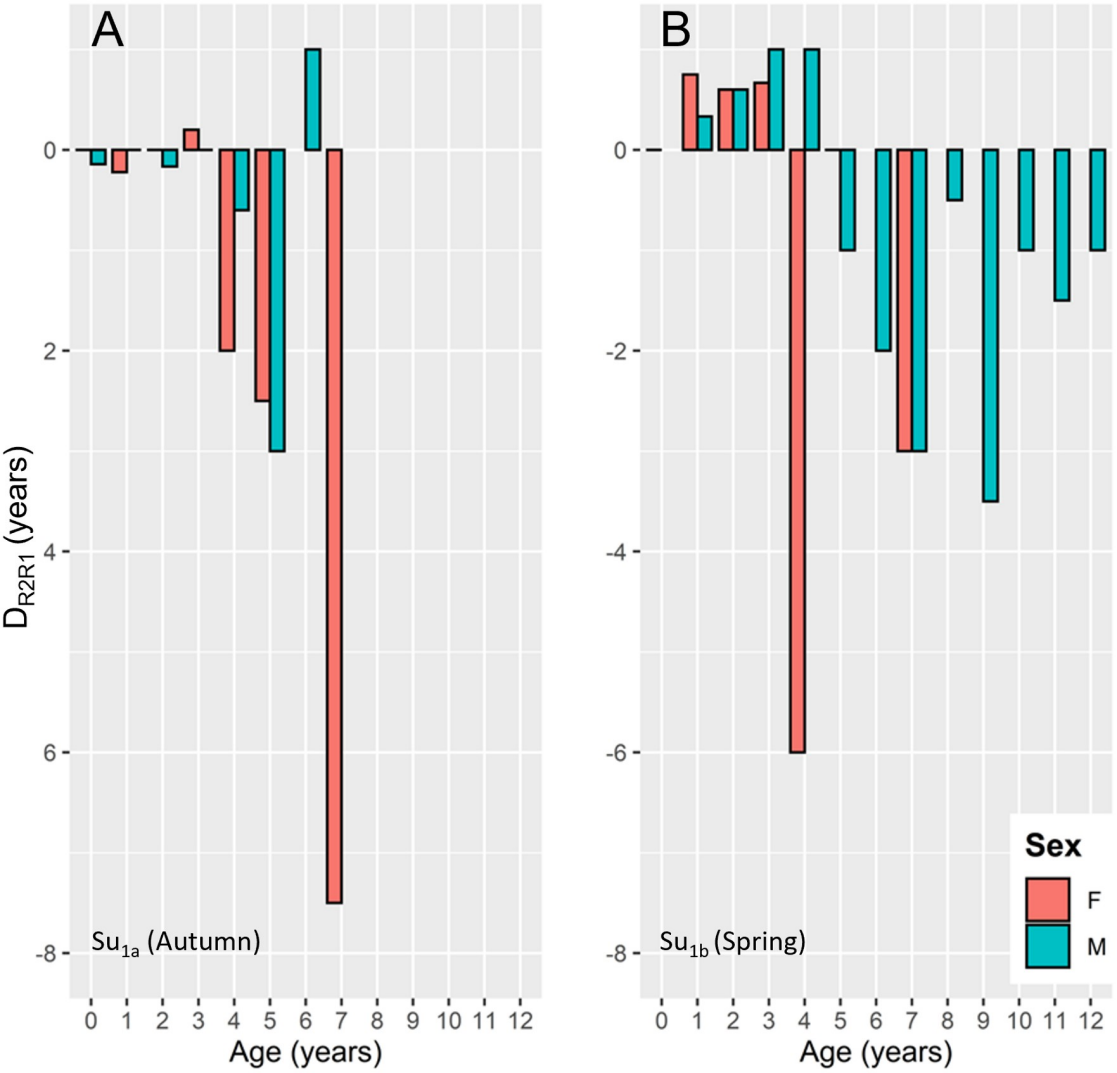
Differences in mean deviation between R1_GLG_ and R2_Stdg_ (D_R1R2_) in Norwegian harbour porpoises across sample units. (A) Su_1a_ (Autumn 2016) and (B) Su_1b_ (Spring 2017).

More detailed analyses based on the average deviance between R2_Stdg_ and R1_GLG_ (D_R2R1_) showed a clear shift in magnitude and direction of D_R2R1_ for young animals between sample units (Fig 2). Because the variance of the deviations increased markedly from R1_GLG_ ≥4, statistical analyses were run separately for animals with R1_GLG_ up to three years (N = 81) and older animals (N = 23). Candidate explanatory variables were sex, sample unit and R1_GLG_, which were tested in GAMs with normally distributed errors. The best model for the young samples included only a factor for sample unit (ΔAIC_icpt_ = -29.5).

### Sex ratio/ male proportion

The overall foetal sex ratio in the present study was exactly 1:1, while the postnatal sex ratio was 1.3:1 (Table 1). The postnatal male proportion of 0.57 was not significantly different from 0.5 (binomial test, *p* = 0.14). GAM analysis with binomial errors showed no significant effect of sampling unit or smooth parameters for age based on R1_GLG_ or R2_Stdg_ on the male proportion in the data set.

### Foetal growth and dates of conception and birth

**Fig 3:**
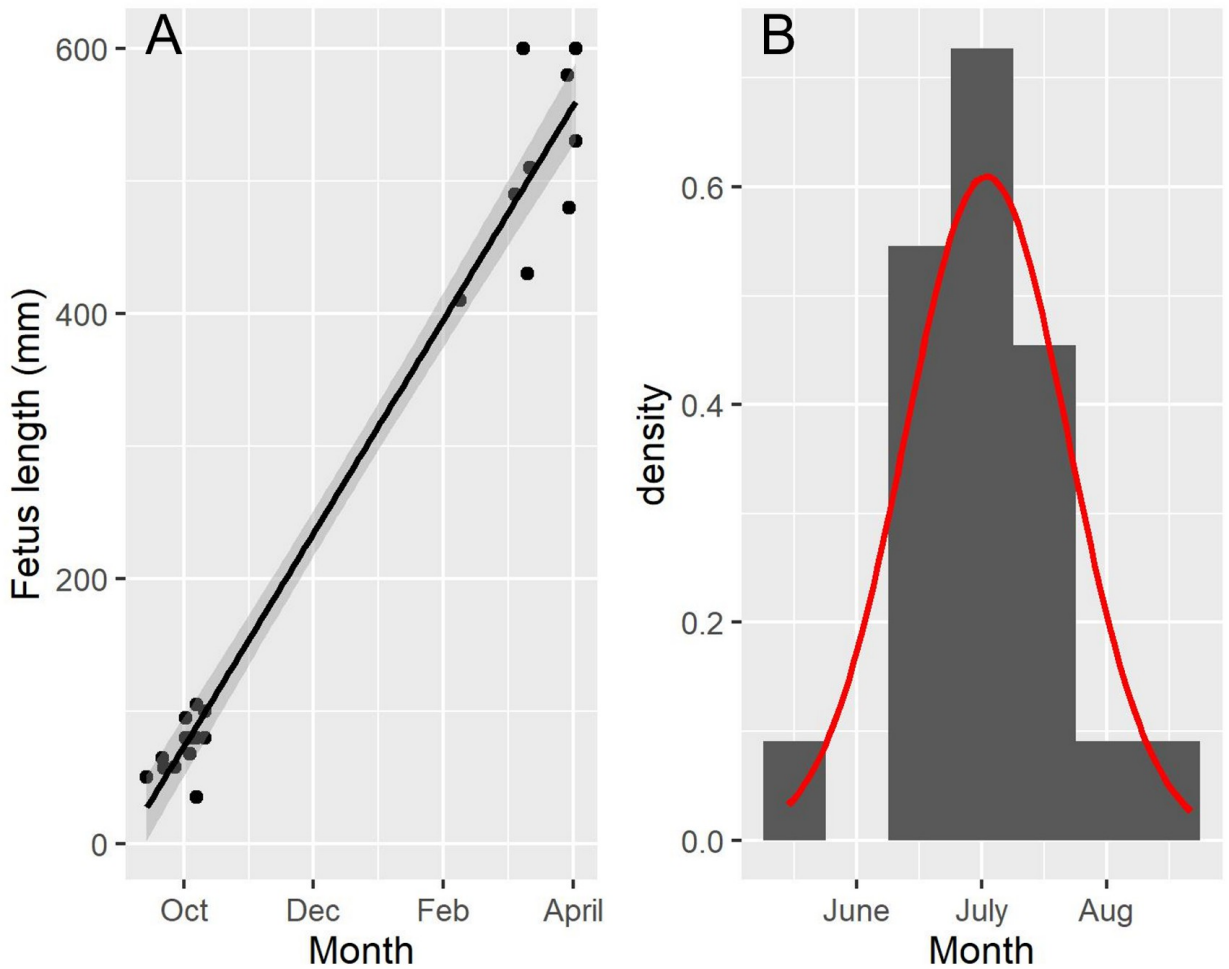
Foetal growth and conception date for Norwegian harbour porpoises. (A) Foetal length as a function of date of death. (B) Back-calculated conception dates for 22 foetuses (see text for details).

The foetal body length range was 24–810 mm (N = 22). The linear regression of foetal length on month provided a good fit (R^2^ = 97.1%, *p*< 0.001) (Fig 3A). The foetal growth rate (u), indicated by the slope of the regression line was estimated at 80.7 mm/month (Fig 3A). Given a mean foetal growth lag phase (t_0_) of 37.9 days, the estimated date of conception of the foetus (N = 22) ranged between 14 May to 21 August with a mean conception date of 1 July (SD = 19.5 days) (Fig 3B). Given a mean birth length of 762 mm, taken from [30], the mean gestation time was estimated at 326 days (ca. 10.7 months), suggesting a peak date of birth around 23 May.

## Postnatal size at age

**Fig 4:**
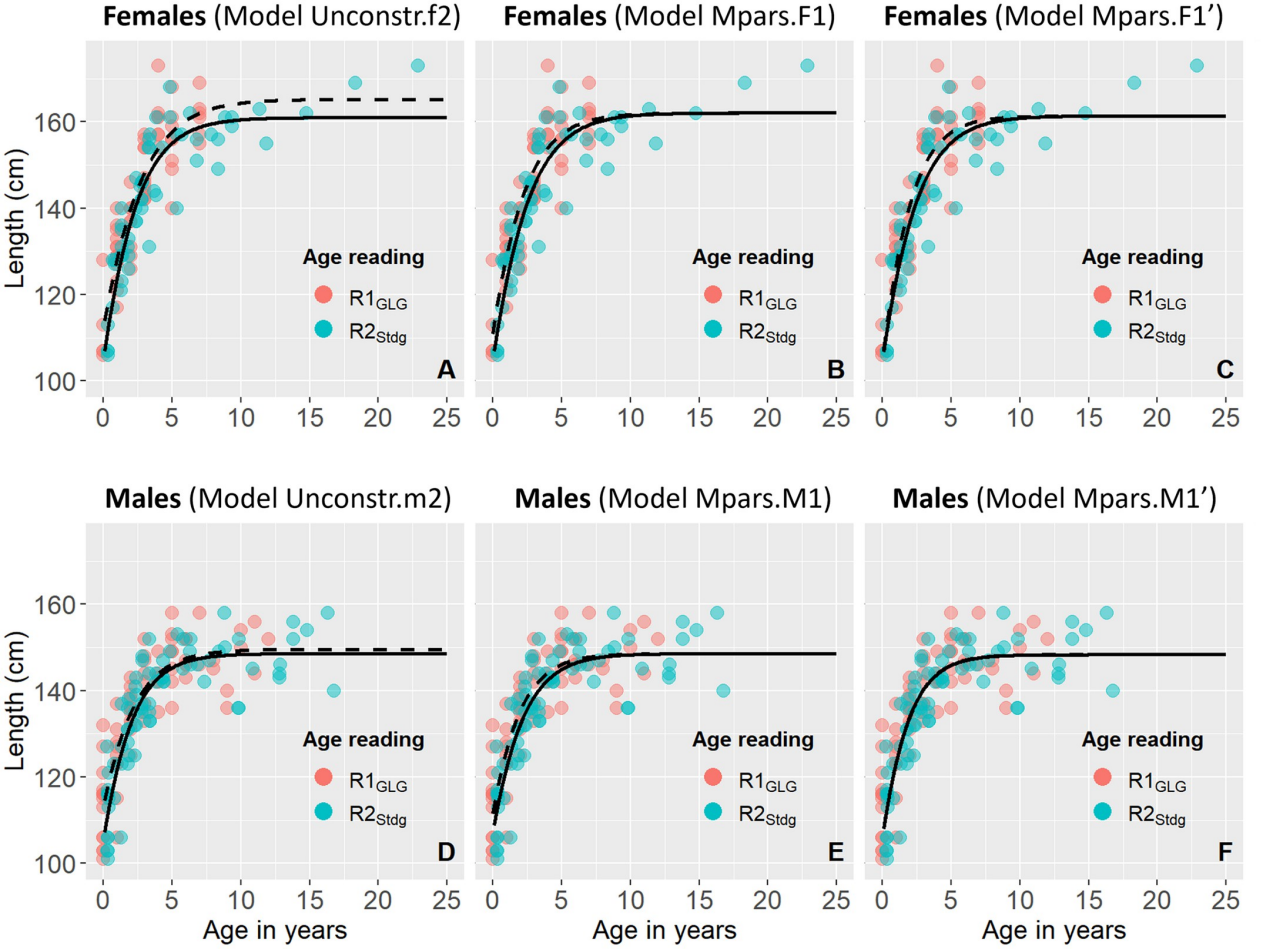
Body length at age for female (A-C) and male (D-F) harbour porpoises from the focal Norwegian data set based on different age reading practices. Lines show the Gompertz growth models indicated by the title above each panel referring to models in Table 2 (females) and Table 3 (males). Dashed lines with orange data points show results for R1_GLG_ and R1_GLG’_. Full lines with cyan data points show results for R2_Decg_ age readings. Ages of observed data points have been skewed by ±0.1 for graphical visibility of both age reading practices.

Table 1 summarizes the mean length and mass of the 134 non-foetus Norwegian focal samples by sex class, sample units and life history stages. Females were generally longer and heavier than males except during the early calf stage (i.e for Su_1a_). For both sexes, average length and weight for each stage was higher for Su_1b_ than for Su_1a_, consistent with a seasonal effect. Effects of sex and sample unit on the length-at age relationship were analysed in GAMs based on R2_Stdg_, R2_Decg_ and R1_GLG_. All three models included a single smooth parameter for age and an additive factor for sex with a highly significant positive coefficient for females of 6.5 cm. Only the model based on R2_Stdg_ retained an additional factor value for sample unit (coeff. Su_1b_ = 3.2cm, *p*<0.05). The model based on R2_Decg_ showed a significantly lower AIC than for both R2_stdg_ (ΔAIC = -8.9) and R1_GLG_ (ΔAIC = -18.3). Further comparisons of R2_Decg_ and R1_GLG_ were conducted separately for each sex based on Gompertz growth curves (Tables 2 and 3, Fig 4).

**Table 2:** Gompertz growth curve parameters for female length-at-age.

Female data and models			Gompertz parameters for female length-at-age models				
AgeRp	Su	Model	L_∞_ (95% CI)	k_0_ (95%CI)	l_0_(95% CI)	LnL	AIC
R1_GLG_	1a	Unconstr.f1	161.1(154.6–167.6)	25.7(16.9–34.5)	108.7(102.8–114.4)	-99.1	206.2
R1_GLG_	1b	Unconstr.f1	171.0(148.8–193.4)	13.1(3.9–22.3)	116.5(105.1–127.9)	-92.7	193.4
R2_Decg_	1a	Unconstr.f1	158.9(153.6–164.2)	27.5(17.02–38.0)	100.3(91.42–109.2)	-103.0	214.0
R2_Decg_	1b	Unconstr.f1	163.7(157.3–170.1)	16.0(8.4–23.6)	111.3(101.5–121.1)	-86.4	180.8
R1+R2	1a+b	Unconstr.f1				Σ-381.1	Σ794.4
R1_GLG’_	1a	Unconstr.f1’	158.3(153.2–163.4)	40.3(22.6–58.0)	97.1(86.4–107.8)	-100.1	208.3
R1_GLG’_	1b	Unconstr.f1’	166.0(152.8–179.2)	19.2(5.4–32.9)	108.2(92.7–123.8)	-90.6	189.2
R1’+R2	1a+b	Unconstr.f1’				Σ-380.1	Σ792.2
R1_GLG_	1ab	Unconstr.f2	165.3(157.2–173.3)	18.5(12.4–24.6)	112.1(104.8–116.0)	-195.4	398.7
R2_Decg_	1ab	Unconstr.f2	161.1(156.7–174.1)	21.9(15.3–28.5)	104.8(97.9–111.7)	-191.1	390.2
R1+R2	1ab	Unconstr.f2				Σ-386.5	Σ780.9
R1_GLG_	1a+b	Mpars.F1	162.1(155.6–168.6)	21.0(14.0–28.0)	111.0(105.2–116.8)	-194.8	
R2_Decg_	1a+b	Mpars.F1	162.1(157.9–166.3)	21.0(14.9–27.1)	105.4(99.0–111.9)	-191.0	
R1+R2	1a+b	Mpars.F1				Σ-385.8	Σ787.5
R1_GLG_’	1a+b	Mpars.F1’	161.4(155.6–167.2)	26.2(16.1–36.3)	104.6(96.4–112.8)	-194.1	
R2_Decg_	1a+b	Mpars.F1’	161.4(157.3–165.5)	21.8(15.3–28.3)	104.6(97.7–111.5)	-191.0	
R1’+R2	1a+b	Mpars.F1’				Σ-385.1	Σ786.0

AgeRp: Age reading practice (R1_GLG_ or R2_Decg_); Su: Sample unit. Su_1a_+_b_ denotes information based on models with separate Gompertz parameters for each sample unit. Su_1ab_ denotes information based on Gompertz parameters fitted to pooled data for Su_1a_ and Su_1b_. Age reading practices are sometimes abbreviated to R1 for R1_GLG_, R2 for R2_Decg_ and R1’ for R1_GLG’_; Model: Imposed constraints on the Gompertz model (“Unconstr.” = no constraints, M_pars_.F = most parsimonious Gompertz parameter configuration for female data based on R2_Decg_ and R1_GLG_, M_pars_.F’ = most parsimonious Gompertz parameter configuration for female data based on R2_Decg_ and R1_GLG’_; *L*_*∞*_ = asymptotic length (cm); *k*_*0*_: growth rate at birth (cm/ year); *l*_*0*_: length at birth (cm); 95% CI:95% confidence intervals; LnL: Log Likelihood of the model (given for each sample unit); AIC: Akaikes Information Criterion (Σ indicates the total AIC for all sample units included in the model).

**Table 3:** Gompertz growth curve parameters for male length- at- age and between-sex comparison.

**Male data and models**			**Gompertz parameters for male length-at-age models**				
AgeRp	Su	Model	L_∞_ (95% CI)	k_0_ (95%CI)	l_0_(95% CI)	LnL	AIC
R1_GLG_	1a	Unconstr.m1	154.3(139.0–169.8)	13.7(6.9–20.5)	113.4(109.3–117.5)	-145.3	298.7
R1_GLG_	1b	Unconstr.m1	147.8(144.8–150.8)	25.8(21.0–30.6)	103.9(86.2–121.6)	-104.7	217.4
R2_Decg_	1a	Unconstr.m1	152.5(144.4–160.6)	15.8(9.6–22.0)	107.8(102.2–112.2)	-141.1	290.2
R2_Decg_	1b	Unconstr.m1	147.6(144.6–150.6)	26.7(8.0–45.4)	99.6(81.7–117.5)	-106.9	221.9
R1+R2	1a+b	Unconstr.m1				Σ-498.1	Σ1028.2
R1_GLG_’	1a	Unconstr.m1’	151.3(140.6–162.0)	19.4(7.8–30.9)	107.4(100.7–114.1)	-145.0	297.9
R2_GLG’_	1b	Unconstr.m1’	147.9(144.8–150.9)	25.6(4.5–46.7)	104.5(86.4–121.7)	-104.3	216.5
R1’+R2	1a+b	Unconstr.m1’				Σ-498.7	Σ1026.5
R1_GLG_	1ab	Unconstr.m2	149.6(145.7–153.5)	15.7(19.9–20.5)	113.0(109.5–116.5)	-252.8	513.5
R2_Decg_	1ab	Unconstr.m2	148.6(145.6–151.6)	19.8(13.9–25.7)	105.6(100.4–110.2)	-249.0	506.1
R1+R2	1ab	Unconstr.m2				Σ-501.8	Σ1011.6
R1_GLG_	1a+b	Mpars.M1	148.5(144.9–152.1)	18.0(12.6–23.3)	111.5(108.0–115.1)	-251.3	
R2_Decg_	1a+b	Mpars.M1	148.5(145.4–151.6)	18.0(12.5–23.7)	107.1(102.3–112.0)	-249.4	Σ1017.4
R1+R2	1a+b	Mpars.M1				Σ-500.7	
R1_GLG_’	1a+b	Mpars.M1’	148.3(139.7–157.0)	21.3(2.7–40.1)	105.9(92.0–120.0)	-250.9	
R2_Decg_	1a+b	Mpars.M1’	148.3(145.3–151.2)	21.3(14.5–28.1)	105.9(100.7–111.1)	-249.8	
R1’+R2	1a+b	Mpars.M1’				Σ-500.7	Σ1015.5
**Between-sex comparison**			**Gompertz parameters for length- at- age models**				
Sex (R2_Decg_)	Su	Model	L∞ (95% CI)	k_0_(95% CI)	l_0_(95% CI)	LnL	AIC
F	1a+b	Mpars.MF1	161.6(157.4–165.8)	21.1(15.0–27.2)	105.1(98.8–111.6)	-191.0	425.2
M	1a+b	Mpars.MF1	148.0(145.2–151.0)	21.1(14.9–27.3)	105.1(100.4–109.8)	-250.0	552.9
F+M	1a+b					Σ-441.0	Σ895.4

All codes and abbreviations are the same as in Table 2 except for changed sex-specific suffixes (m for unconstrained male models, M for most parsimonious male models and MF for most parsimonious models for both female and male samples).

Parameters of unconstrained Gompertz models for length-at-age of both sexes showed substantial nominal differences between sample units. For both sexes, larger values of l_0_ were associated with larger L_∞_, lower k_0_ and vice versa. For both age reading practices, the largest values of l_0_ were observed for Su_1b_ for females and for Su_1a_ for males. Generally, l_0_ and L_∞_ values were larger for the R1_GLG_ readings, particularly for females. Fitting identical Gompertz models to data from both sample units for each reading practice significantly reduced AIC for both males (ΔAIC = -5.9) and females (ΔAIC = -3.2). AIC could be further reduced by constraining L_∞_ and k_0_ but not l_0_ to be equal between reading practices (see models Mpars.F1 and Mpars.M1 in Tables 2 and 3, respectively). Fitting identical Gompertz models to both readings from each sample unit also significantly reduced AIC for males (-3.4), but not for females (0.9) (model not shown). Adding the seasonal correction factors used by R2_Decg_ to the animals aged zero years by R1_GLG,_ substantially reduced the differences in Gompertz parameters between reading practices for the same sample units (Tables 2 and 3, AgeRp = R1_GLG’_). With this modification, the most parsimonious Gompertz model for females initially fitted identical parameters to each sample unit across reading practices (AIC = 789.9). A further reduction in AIC was thereafter achieved by fitting common L_∞_ and l_0_, but not k_0_ between reading practices (ΔAIC = -3.8, Model = Mpars.F1’, Table 2). For males, alternative initial models fitting common curves within reading practices and seasons, respectively, produced exactly the same highly significant reduction in AIC (-9.4). The most parsimonious model fitted identical parameters across both reading practices and sample units (Model = Mpars.M1’, Table 3). Direct comparisons of Gompertz parameters for length-at-age between sexes based on R2_Decg_ also showed no significant differences in l_0_ (105.1cm) or k_0_ (21.1 cm/year) but a highly significant difference in L_∞_ of ~13.6 cm (Table 3). Constraining all Gompertz parameters to be equal for both sexes would increase AIC by 39.7 units (not shown).

### Male reproductive parameters

**Fig 5:**
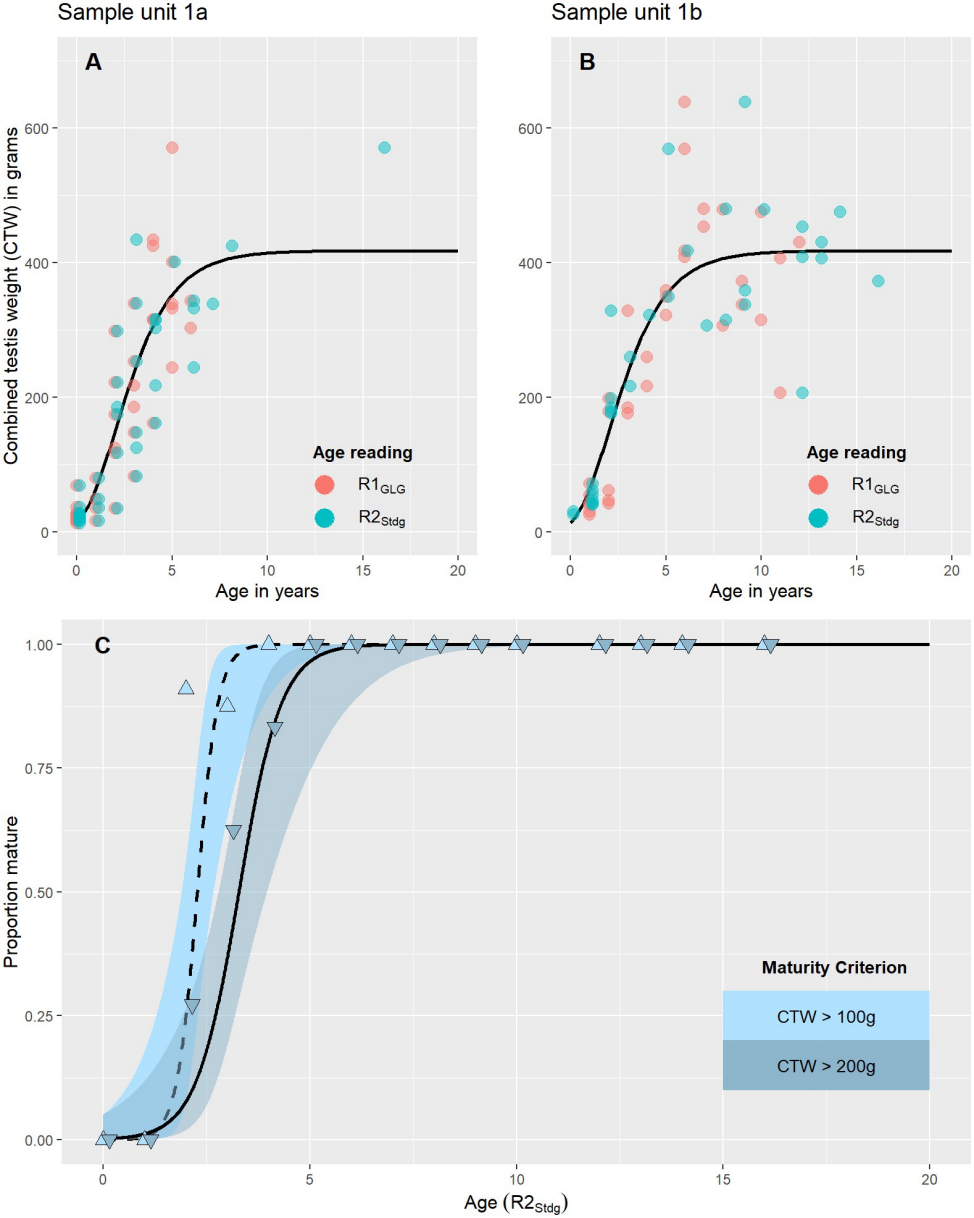
Age-specific male reproductive status for the focal Norwegian data set across sample units, age reading practices and maturity criteria. (A) and (B) Combined testis weight (CTW) as a function of age based on R1_GLG_ and R2_Stdg_ for sample unit 1a and 1b, respectively. The curves show the most parsimonious Gompertz model, which is identical across sample units and age reading practices. (C) Age-specific proportions mature based on two different CTW maturity criteria. Fitted curves are the most parsimonious curves for the two maturity criteria as shown in Table 5 (only shown for R2_Stdg_).

For the autumn samples of the focal Norwegian data set (Su_1a_), all males younger than two years (based on both reading practices) had a CTW lower than 100g (Fig 5A).

A gradual increase in CTW was seen in the older age classes, along with an apparent increase in variance. Only two males older than one year had a CTW <100g (Fig 5A and 5B). For spring samples, all CTWs in R2_Stdg_ age classes >1 year were close to 200 g or more, whereas two individuals with R1_GLG_ >1 year had a CTW <100g (Fig 5B). Both individuals were assigned an R1_GLG_ age of two years, based on a newly formed boundary layer, but were likely only approaching their second summer. Interestingly, none of the males approaching two years in spring (i.e. R2_Stdg_ = 1) had a CTW>100g (Fig 5A), whereas several two-year-olds in the autumn samples had CTW >100g and even CTW>200g (Fig 5B). For all seasons and readings, CTWs appeared to stabilise at an average of about 400 g around five to six- years of age. No CTW<200g was observed over the age of five years for any of the age reading practices or seasons. GAMs fitted to all the CTW data showed no significant effect of factor variables for age reading practice or sample unit, but a highly significant nonlinear effect of age (ΔAIC_itcpt_ = 203.4). Fitting a joint Gompertz growth curve to all the CTW-at-age data further reduced AIC by 4.6 units (curve shown in Fig 5A and 5B).

For CTW_100g_, unconstrained Richards maturity curves showed values of MAM ranging from 2.0 years for Su_1b_ based on R2_Stdg_ to 2.6 years for Su_1b_ based on R1_GLG_ (Table 4).

**Table 4:** Life history parameters of male harbour porpoises with methodological metadata.

Sample unit (AgeRp)	Sample source (N_R_; N_G_)	Smp_per_	sRC	sAgeCrit	A_mx_	Mat_crit_	MAM	ASM_SOFI_	A_50_	L_∞_
				R/G			Unc		Log; Unc	
**1a** Norw.*(R1*_*GLG*_*)*	B (41;42)	16	EG	GLG/GLG	6	CTW_100g_	2.2±	2.3±	1.8;1.5	154.3
**1b** Norw.*(R1*_*GLG*_*)*	B (34;34)	17	LG	GLG/GLG	12	CTW_100g_	2.6	2.6	2.0;2.1	147.8
**1a** Norw.*(R2*_*Stdg*_*)*	B (41;42)	16	EG	Stdg/Decg	16	CTW_100g_	2.4	2.3	1.8;1.5	152.5
**1b** Norw.*(R2*_*Stdg*_*)*	B (34;34)	17	LG	Stdg/Decg	16	CTW_100g_	2.0	-	1.4;1.1	147.6
**1ab** Norw.*(R2*_*Stdg*_*)*	B (75;76)	16–17	EG/LG	Stdg/Decg	16	CTW_100g_	2.4	2.3	1.4;1.1	148.5
**1c** Norw.	B (70;74)	88–90	PN/All	GLG/GLG	8	CTW incr.	2–3	-	-	142.3±4.1
**2a** Icelnd	B (>500)	91–97	LG/EG	Stdg/Decg?	16	Mix. Hist.	2.6 ±0.2	-	1.9±?	149.5± -
**3a** Scotlnd	B/S (141;111)	92–05	All	GLG^+^/GLG^+^	20	Mix. Hist.	-	-	5.0 ±1.7	149.7±2.6
**3c** Dnmrk	B/S (102; -)	85–91	All	GLG/GLG?		Mix. Hist.	-	2.9±?	-	-
**3d** Dnmrk	B/S/H (135;338)	1838–1998	All	GLG/GLG?		CTW_200g_	-	3–4	-	145± -
**3g** SE.UK	B/S (62;83)	90–99	All	GLG’/GLG	18	Mix. Hist.	-	-	3.6±0.5	140.9±3.3
**3h** SE.UK	B/S (45;49)	00–12	All	GLG/GLG’	15	Mix. Hist.	-	-	3.6±0.5	140.9±3.3
**5a** SW.UK	B/S (78;109)	90–99	All	GLG/GLG	18	Mix. Hist.	-	-	3.6±0.5	146.5±3.2
**5b** SW.UK	B/S (66/83)	00–12	All	GLG/GLG’	15	Mix. Hist.	-	-	3.6±0.6	146.5±3.2
**3gh+5ab** Total UK	B (47’;-)	90–12	All	GLG/GLG	5’	Mix. Hist.	2.7^r^	3.5	2.2*;1.5^r^	-
**6a** B.Biscay	B/S (40;162)	90–10	All	GLG/GLG	19	Mix. Hist.	-	-	3.8± -	162± -
**7a** W.Grl	B (39;81)	88–95	PN/EG	GLG/GLG	17	CTW_200g_	2.5 ±?	-	-	142.8±3.5
**8a** E.Nfd	B (59;59)	90–91	PN	GLG/GLG	12	±Sperm	3.0±0.2	-	-	142.9±2.4
**9a** B.Fndy	B/H (-;56)	69–73	EG/PN	GLG/GLG	10	-	-	-	-	146.0±4.1
**9b** B.Fndy	B/H (-;121)	85–88	EG/PN	GLG/GLG	10	-	-	-	-	144.0±3.5
**9c** Maine	B (31,-)	89–93	All	GLG/GLG	15	Mix. Hist.	-	3–4	-	-

Codes for sample units refer to Fig 1 (see also material and methods section for reference studies) and are supplemented with abbreviated approximate area names (SE.UK = Southeast UK, SW.UK = Southwest UK).Separate results are given for each reading practice for the focal Norwegian data set.; Sample source: B = bycatch, H = hunting, S = strandings; N_R_/N_G_: Numbers of individuals with reproductive data (N_R_) and/or length data (N_G_); Smp_per_: Last two digits of first and last year of sampling period (no sampling prior to 1969); sRC: Reproductive phases included in sampling period (PN = perinatal period from calving or first ovulation to implantation, EG = early gestation, LG = late gestation); sAgeCrit: Seasonal age assignment criterion for reproductive (R) and growth (G) samples (see methods section for definitions); A_mx_: Maximum age in sample; Mat_Crit_: Criterion for sexual maturity: CTW incr. = the approximate age span of rapid CTW increase, Mix.Hist. = mixed histological criteria, CTW_200g_ and CTW_100g_ indicate threshold CTWs as explained in text; for MAM, ASM_SOFI_, A_50_ and L_∞_, see text. “Unc.” and “Log.” denote calculations based on an unconstrained Richards curve and a logistic curve, respectively; ^r^: parameters reanalysed for the present paper; symmetric upper and lower 95% confidence limits are indicated where available; -: Not available/unknown.

The ASM_SOFI_ for unsmoothed proportions immature were within one decimal of MAM in three of four comparisons based on unconstrained models (Table 4) due to good fits of the Richards curves. ASM_SOFI_, cannot, however, be estimated for samples with knife-edge recruitment as seen for Su_1b_ based on R2_Stdg_ ages (Fig 5B, Table 4). The most parsimonious Richards curve estimated a MAM of 2.4 years and an A_50_ of 2.0 years (Table 5). This Richards curve had a shape parameter (*m*) of zero (not shown) and is thus in effect a von Bertalanffy curve. Imposing a Gompertz curve (*m* = 1) increased AIC by 0.6, while imposing a logistic curve (*m* = 2) increased AIC by >5 in comparison (not shown).

**Table 5:** Estimates of age and length at maturity for Norwegian harbour porpoises based on the most parsimonious Richards models.

Sex	Data	Su	Mat_crit_	AgeRp	MAM	A_50_	L_50_
					(95% CI)	(95% CI)	(95% CI)
M	Norway	Su_1a+b_	CTW_100g_	R1_GLG_	2.4 (2.2–2.6)	2.0 (1.8–2.3)	135.3 (125.6–144.3)
		Su_1a+b_	CTW_100g_	R2_Stdg_	2.4 (2.2–2.6)	2.0 (1.8–2.3)	132.6 (121.4–142.7)
		Su_1a+b_	CTW_200g_	R1_GLG_	3.4 (3.1–3.8)	3.2 (2.9–3.8)	139.5 (131.2–149.2)
		Su_1a+b_	CTW_200g_	R2_Stdg_	3.4 (3.1–3.8)	3.2 (2.9–3.8)	137.3 (127.5–148.2)
M	Lit. Range	Su_3h_; Su_2a_	Mix. Hist.				129.5±1.3–135±NA
F	Norway	Su_1a_	CL/CA/foetus	R1_GLG_	3.2 (3.0–3.5)	2.9 (2.4–3.0)	148.3 (130.9–160.1)
		Su_1a_	CL/CA/foetus	R2_Stdg_	3.2 (3.0–3.5)	2.9 (2.4–3.0)	144.9 (127.3–156.1)
		Su_1b_	CL/CA/foetus	R1_GLG_	4.0 (3.6–4.6)	3.5 (3.1–4.1)	151.8 (135.8–164.4)
		Su_1b_	CL/CA/foetus	R2_Stdg_	4.0 (3.6–4.6)	3.5 (3.1–4.1)	148.9 (133.0–161.0)
		Su_1a+b_	CL/CA/foetus	R2_Stdg_	3.4(3.0–4.0)	2.9 (2.5–3.5)	144.9 (128.2–158.7)
F	Lit.Range	Su_3g+h_; Su_2a_	CL/CA/foetus				138.91.5–146±NA

Estimates of mean age at maturity (MAM), average age and length of 50% maturity (A_50_ and L_50_) estimates for males (M) and females (F) in the present data set and with sex-specific ranges of literature values for the Northeast Atlantic. Su refers to the sampling units in Fig 1. Mat_crit_ indicates the criteria for maturity (abbreviations for males are explained in Table 4 legend). Confidence intervals of L_50_ are derived by entering the 95% confidence intervals of A_50_ into length at age curves based on the corresponding confidence intervals of Gompertz parameters.

For the CTW_200g_ maturity criterion, estimates of MAM based on unconstrained Richards curves ranged from 2.8 years (R2_Stdg,_ Su_1b_) to 3.7 years (R1_GLG,_ Su_1b_) (not shown). The most parsimonious Richards curve estimated a common MAM of 3.4 years and an A_50_ of 3.2 years for all sample units and age reading practices (Table 5). The lowest AIC was obtained with a shape parameter very close to one (effectively a Gompertz curve) but imposing a logistic curve or a von Bertalanffy curve only increased AIC by less than one. Due to between-reader differences in length-at-age models, there were minor differences in point estimates of L_50_ between reading practices (Table 5).

To increase comparability between studies, MAM and A_50_ based on fitted Richards curves were also estimated for published data from UK waters previously analysed by [19] (see Table 4). Using only data for trauma-killed males, the observed proportions mature for two- and three-year-olds were 0.85 and 0.14, respectively, displaying a clear deviance from the generally expected sigmoid shape of the age-related maturation process. None of the older age classes reached full maturity. A completely unconstrained Richards maturity curve estimated the proportion mature among zero-year-olds at 0.69. Estimates of MAM and A_50_ given in Table 4 are based on models constrained to estimate the proportion mature among zero-year-olds at <0.01. The resulting estimate of MAM of 2.7 years is almost one year lower than the ASM_SOFI_ value reported by [19]. The best-fitting maturity curve for this sample was a von Bertalanffy curve. Imposing a logistic curve reduced the overall AIC by >30 and changed A_50_ from 1.5 years to 2.2 years.

### Female reproductive parameters

#### Features of female reproductive stages in the focal Norwegian data set

**Fig 6:**
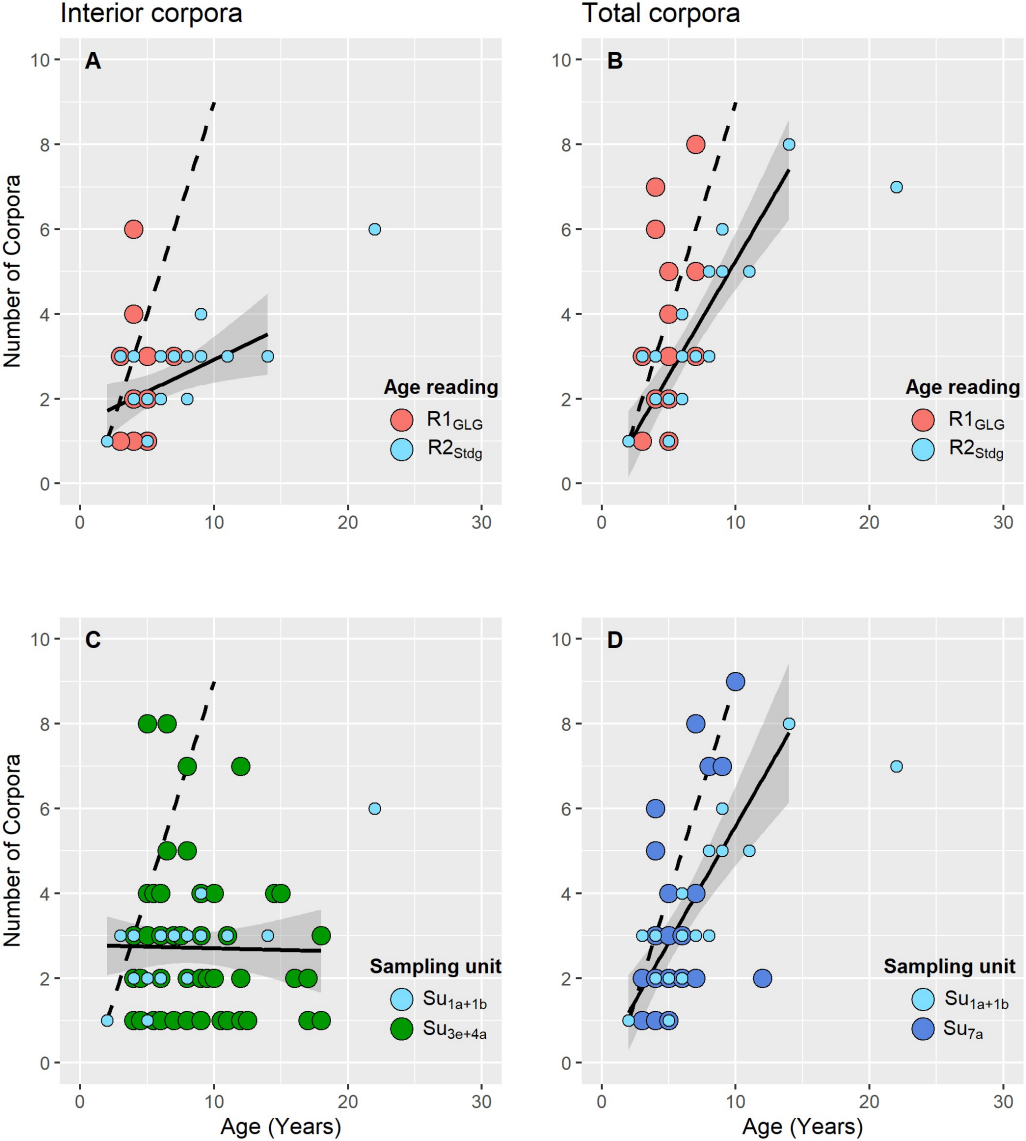
Accumulated numbers of interior ovarian *corpora* (left panels) and total ovarian *corpora* (right panels) in mature harbour porpoise females from Norway, Germany and Greenland. Upper panels show results by age reading practice for the focal Norwegian data set. Lower panels show results by sample unit(s) for data sets of various origins (only R2_Stdg_ based results shown for Norwegian data). Su_1a_+Su_1b_ denotes the combined sample units of the focal Norwegian data set (N = 19 mature females). Su_3e_+Su_4a_ are combined German sample units for the North Sea and Baltic Sea from [61] (N = 59 mature females). Su_7a_ is West Greenland data presented by [52] (N = 31 mature females). Dotted lines show the expected number of *corpora* accumulated assuming annual formation of a single persistent corpus since the second year of age. Solid lines show either the optimal GAM for R2_Stdg_ (upper panels) or the optimal joint model for all sample units (lower panels).

The youngest mature female in the focal Norwegian data set was caught in spring 2017 and was assigned an R1_GLG_ age of three years and an R2_Stdg_ of two years (Fig 6A and 6B). She did not have a foetus but did have one CA which was visible inside the ovary (“interior CA”). This female was 142 cm long and was the second smallest of the mature females.

The smallest mature female was 140 cm long and her age was estimated at five years by both age reading practices. The lengths of non-calf immature females with no sign of ovarian *corpora* ranged from 121 cm to 161 cm. The second-largest immature female was only 147 cm long and the maximum immature length therefore seems to be an outlier. The largest and oldest immature female was bycaught in late March and was assigned an R1_GLG_ of four years and an R2_Stdg_ of three years. She was 161 cm long and had smooth uterine horns consistent with a nulliparous status. Three other immature females were assigned an R2_Stdg_ of three years. Assessments of parity status of the uterus were available for 21 females from Su_1b_ with associated ovary data. Six of these uteri were characterised as mature and all of these had a foetus and at least one interior CA (three had additional surface CAs). One female with an immature looking uterus had three interior CAs but no foetus. She was aged four and five years based on R2_Stdg_ and R1_GLG_, respectively.

Among the six oldest females based on R1_GLG_ (all seven years old), five were pregnant. Two of these had missing ovary data, while the last three had two interior CAs and a CL. Ovary data were also missing for the non-pregnant female with R1_GLG_ = 7. With a standard length of 169 cm this female was the second-largest mature female in the focal Norwegian data set. She was estimated at 18 years by reader R2, who also estimated nine other females to be more than seven years old. The lengths of these females ranged from 149 cm to 173 cm. The largest was the previously mentioned female estimated to be 22 years old by R2_Stdg_ and four years by R1_GLG_. She had five interior CAs, one surface CA, a CL and a foetus.

#### Corpora accumulation with age in the focal Norwegian data set and two other data sets

For the focal Norwegian data set, both total and interior ovarian *corpora* accumulated with age for both age reading practices (see Fig 6, upper panels). GAM analyses based on the 19 females with at least one ovarian corpus (CA or CL), showed a highly significant (*p*<0.001) linear increase (not shown) in the number of superficial CAs for all (R1_GLG_) or most of the age range (R2_Stdg_). In the latter case, the single superficial CA in the oldest female of 22 years, enforced a declining trend from age ~15 years. If this individual was left out, the best model included a common linear relationship for age based on both reading practices (*p*<0.01). The distinction between superficial and interior CAs did not affect the assigned maturity status for any of the females although one gestating female aged four years by R1_GLG_ and five years by R2_Stdg_ only had a superficial CA and therefore would have been classified as immature if she had not had an active CL. Only one female with a CL did not also have a CA. This female was caught in October 2016 and was estimated to be five years old by both age reading practices.

Interior CAs occurred from two years of age for R2_Stdg_ and from three years for R1_GLG_ ages, while superficial CAs occurred from four to five years of age for R1_GLG_ and R2_Stdg_, respectively. For R1_GLG_ ages, back-calculated ages of first ovulation< 2 years were observed for three females based on interior CAs (-1 to 1 years) and for six females based on total CAs (-2 to 1 years). The effect of CA classification practice was however not statistically significant. Only one female in this data subset did not have at least one surface CA. The maximum individual number of excess CAs for R1_GLG_ was three based on interior CAs and four based on total CAs. For R2_Stdg_, three females aged two to four years at death had an unrealistically low back-calculated age at maturity of one year. None of these females had any surface CAs and the maximum number of excess CAs was one. For R1_GLG_ ages, unrealistically high back-calculated ages at maturity were seen for three seven-year-old females based on interior CAs (in all cases five years). Due to additional surface CAs, two of these females had back-calculated ages at maturity <5 years based on total CAs. Based on R2_Stdg,_ unrealistically high back-calculated ages at maturity were seen in 10 cases for interior CAs (5–17 years) and seven cases for total CAs (5–16 years). For the R2_Stdg_ data subset, a binomial GAM for the proportion of unrealistically high ages at maturity, showed a significant linear effect of age (ΔAIC_itcpt_ = 23.4). There was no significant additive effect of CA classification practice (ΔAIC = -0.9).

Comparisons of accumulation patterns for interior and total *corpora* counts for the focal Norwegian data set were conducted separately for each age reading practice. A significant linear effect of age was found in all cases except for the combination of R2_Stdg_ and total *corpora* counts, which showed an asymptotic pattern induced by the oldest female of 22 years. After excluding this female, models for R2_Stdg_ showed a highly significant interaction term (*p*<0.001) between age and *corpora* classification practice with slopes ranging from 0.15 (±0.14, 95% CI) interior *corpora*/year to 0.54 (±0.48, 95% CI) total *corpora*/year (Fig 6A and 6B, respectively). The intercepts varied from 1.42 (±0.96, 95% CI) for interior CAs to -0.14 (±2.28, 95% CI) for total CAs (*p*<0.05). The best model for R1_GLG_ (not shown) had a significant (*p*<0.01) uniform slope of 0.53 (±0.38, 95% CI) CAs/year and a marginally significant additive intercept coefficient of 0.94 (±0.86, 95% CI) for total CAs compared to -0.19 (±2.0, 95% CI) for interior CAs. No effects of sample unit or sampling season/year were found for any of the age reading practices.

Direct comparisons with previously published age-specific *corpora* counts from West Greenland and Germany were performed by adding these data sets to the Norwegian data sets for interior and total CAs and treating all four data sets as separate CA classification practices. During the initial organisation of the German data set, it was noted that simultaneous presence of two CLs had been recorded for two females among a total of 39 females with a CL. The other 37 females only had one CL. The double CLs were observed in females stranded in late November and early December, several months after the end of the breeding and implantation season. No cases of multiple CLs were recorded in the focal Norwegian data set and no cases were reported for the West Greenland data set either [52].

Statistical analyses across the Norwegian, German and Greenlandic data sets were conducted separately for Norwegian data based on R1_GLG_ and R2_Stdg_. The female aged four and 22 years, respectively by the two age reading practices was excluded from these analyses. For both Norwegian age reading practices, the best GAM comprised separate intercepts and age smooths for all four data sets (ΔAIC<-14). All the estimated age smooths were linear but only the smooths for Norwegian total *corpora* counts and West Greenland *corpora* counts were significant in this analysis (*p*<0.001 to *p*<0.05). Highly significant differences were found between the German and the West Greenland data sets for both intercept and slope (*p*<0.001). The estimated slope for the West Greenland data set was 0.78 ± 0.28 (0.95 CI) *corpora*/year while the nominal slope for the German data set was -0.05± 0.12 (0.95% CI). The slope and intercept for Norwegian total *corpora* counts also differed significantly from the German data set based on both R1_GLG_ (*p*<0.05) and R2_Stdg_ (*p*<0.001). The corresponding slopes ranged from 0.82 ± 0.84 (0.95% CI) for R1_GLG_ to 0.84 ±0.40 (0.95% CI) for R2_Stdg_. The estimated nominal slopes for the Norwegian interior *corpora* ranged from 0.15 ±0.40 (0.95% CI) for R2_Stdg_ to 0.25 ±0.84 (0.95% CI) for R1_GLG_. The model based on R2_Stdg_ showed a significantly better fit to the data than the R1_GLG_ based data (ΔAIC = -11). Treating the German data and the Norwegian interior *corpora* data as one unit and the remaining two data sets as another unit further reduced overall AIC by 1.8 for R2_Stdg_ and by 5.1 for R1_GLG_. The model based on R2_Stdg_ still had an overall lower AIC than the model based on R1_GLG_ (ΔAIC = -7.7) and was chosen for display in Fig 6C and 6D. The optimal model expressions were 2.78 (±0.92) -0.01 (±0.92) *Age for the first unit (Fig 6C, solid line) and -0.28 (±1.20) + 0.63 (±0.92) *Age for the second unit (Fig 6D, solid line). Both intercept and slope differed strongly between units (*p*<0.0001).

#### Age at female maturity and pregnancy rates for the Norwegian focal data set

**Fig 7:**
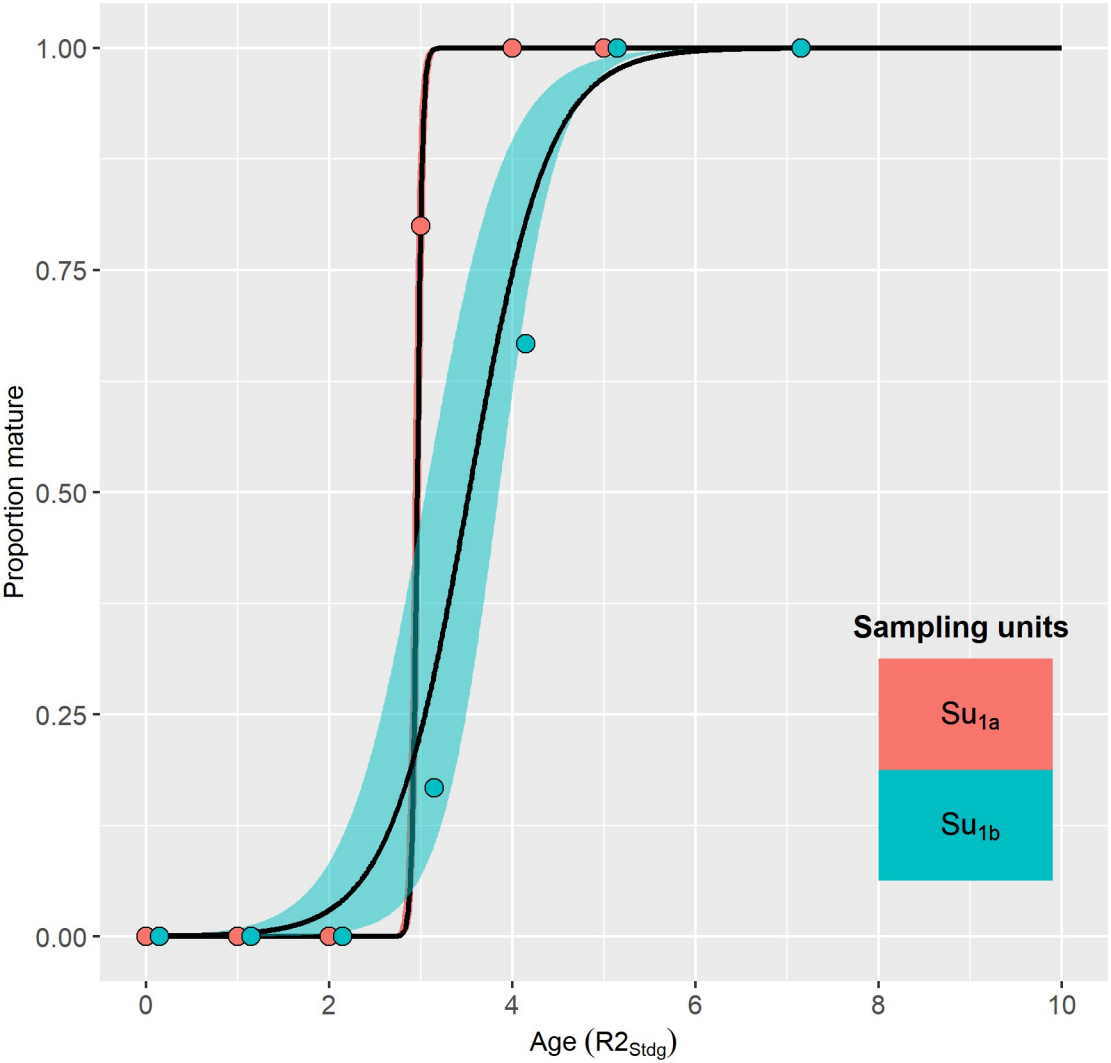
Female age-specific proportions mature for the focal Norwegian data set based on R2_Stdg_ ages. The curves show the most parsimonious Richards functions for Su_1a_ and Su_1b_ (see parameters in Table 5).

The analyses of age at maturity were based on 55 females (28 from Su_1a_ and 27 from Su_1b_) with a sufficient reproductive record to document signs of previous ovulation (CL, CA or foetus). Unconstrained Richards maturity curves were fitted to each of the four combinations of sample unit and age reading practice (Table 6, Fig 7). MAM was estimated at 3.2 years for Su_1a_ based on both age reading practices but differed by 0.5 year between readings for Su_1b_ (R1_GLG_ = 4.2 years, R2_stdg_ = 3.7 years). Estimates of A_50_ were 0.2–0.3 years lower than MAM values while estimates of MAM_SOFI_ were up to 0.9 years lower than MAM (Table 6). Separate tests for each reader showed significant differences in MAM between seasons for R1_GLG_ (ΔAIC = -5.9) but not for R2_Stdg_ (ΔAIC = -1.7). The most parsimonious overall Richards model (AIC = 41.0) fitted identical Richards curves to data for both age reading practices within seasons (Table 5). AIC was further reduced by -2.9 after imposing a logistic model (*m* = 2). This model estimated MAM at 3.2 and 4.0 for Su_1a_ and Su_1b_, respectively and A_50_ values 0.3–0.5 years lower (Table 5). Forcing *m* = 2 further reduced AIC by 1.9 (see Table 6). The estimated MAM and A_50_ of this model were 3.6 years and 3.1 years, respectively.

**Table 6:** Life history parameters of female harbour porpoises with methodological metadata.

Sample Unit *(AgeRp for R)*	Sample Source/COD (N_R_; N_G_)	Smp_per_	sRC	sAgeCrit	A_mx_	PR_mat_; N_Mat_	MAM	ASM_SOFI_	A_50_	L_inf_
				R/G			Unc		Log; Unc	
**1a** Norw.*(R1*_*GLG*_*)*	B (28;31	‘16	EG	GLG/GLG	7	100; 14	3.2±0.3	3.8±0.1	2.9;2.9	161.1
**1b** Norw.*(R1*_*GLG*_*)*	B (27;27)	‘17	LG	GLG/GLG	7	73.0; 11	4.2±0.8	4.2±0.3	3.6;3.9	171.4
**1a** Norw.(R2_Stdg_)	B (28;31)	‘16	EG	Stdg/Decg	11	100.0;14	3.2±0.3	3.8±0.1	3.0;2.9	158.9
**1b** Norw.*(R2*_*Stdg*_*)*	B (27;27)	‘17	LG	Stdg/Decg	22	73.0;11	3.7±0.8	2.2±0.1	3.3;3.9	163.7
**1ab** Norw.*(R2*_*Stdg*_*)*	B (55;58)	16–17	EG/LG	Stdg/Decg	22	88.0;25	3.4±0.8	3.7±0,1	3.1;3.1	162.1
**1c** Norw.	B (56;56)	88–90	All {PN}	GLG/GLG	8	{36.4^c^;11}	4.0^r^	4.1± 0.2	3.9^r^;4.9^r^	161.0±18.8
			All {PN}			{33.3; 9}				
**2a** Icelnd	B (>200)	91–97	LG/EG	Stdg/GLG^+^	20	98.6;74	4.4±0.6	4.2^r^	3.2±?	160.1± -
**3a** Scotlnd	B+S (144;144)	92-‘05	All	GLG^+^/GLG^+^	20	27.6;76	-	-	4.4±0.8	163.0±4.4
	B+S	92-‘05	-PN			40.4;42				
**3b** W.Swd.	B (78;60)	88–90	All	GLG/GLG	8	71.4^c^;21	4.4^r^	4.3± 1.0	3.8^r^;4.1^r^	158.2±4.2
			All			67.7;18				
**3c** Dnmrk	B+S (83;-)	85–91	All	GLG?/GLG	-	61.4^c^;44	-	3.6±?	-	-
			-PN			72.2;33				
**3d** Dnmrk	B+S (-;?)	~62–98	All	GLG/GLG?	23	-	-	-	-	160± -
**3e** Gm.NS	B+S (111;-)	87-‘16	All	GLG++/-	18	-	-	-	5.0±0.6	-
**3f** Dtch.NS	B+S (154;-)	‘06-‘19	All	GLG+/	24	28.3;180	-	-	4.0±0.5	-
	B+S		-PN			34.5;161				
	T		-PN			58.0; 38				
**3g** SE.UK	B+S (68;72)	90–99	All{-PN}	GLG/GLG	22	{26.3;19}	-	-	3.8±0.5	155.4±4.0
**3h** SE.UK	B+S (49;51)	‘00-‘12	All{-PN}	GLG/GLG’	15	{30.4;23}	-	-	4.8±0.6	155.4±4.0
**4a** Gm. BS	B+S (111;-)	87-‘16	All	GLG++/-	18	-	-	-	5.0±0.6	-
**5a** SW.UK	B+S (102;104)	90–99	All{-PN}	GLG/GLG	15	{68.0;25}	-	-	3.8±0.5	162.9±4.0
**5b** SW.UK	B+S (86;87)	00-‘14	All{-PN}	GLG/GLG’	21	{54.3;35}	-	-	4.8±0.6	162.9±4.0
**3gh+5ab** Total UK	T (28;-)	90-‘14	All	GLG/GLG	5	-	4.7^r^	4.4	4.1^r^;3.8^r^	-
**6a** B.Bisc.	B+S (48;127)	90–10	All{-PN}	GLG/GLG	18	{53.8;13}	-	-	5.5±?	185± -
**7a** W.Grl	H (>55;84)	88–95	PN	GLG/GLG	14	-	3.6±?	-	-	154.0±5.2
**7a** W.Grl	H (55;-)	95	PN	GLG/GLG	-	-	3.7±0.1	-	-	-
**7b** W.Grl	H (60;-)	‘09	PN	GLG/GLG	-	-	3.5±0.1	-	-	-
**8a** E.Nfd	B (32;33)	90–91	PN	GLG/GLG	9	88.2^c^;17	3.1±1.4	3.1±^r^	2.6^r^;3.0^r^	156.3±6.0
**9a** B.Fndy	B+H (37;44)	69–73	EG/PN	GLG/GLG	9	89.5^c^;19	4.0±0.5	-	-	163.0±8.4
**9b** B.Fndy	B+H (108;116)	85–88	EG/PN	GLG/GLG	14?	86^c^.0;50	3.4±0.4	-	-	155.0±3.5
	B+H (75;-)	85–88	EG	GLG/GLG		74.3;35	-	-	-	-
**9c** Maine	B (99;-)	89–93	EG	GLG/GLG	17	95.0;14	-	3.4± 0.3	-	-
**9d** Mass.	S (18; -)	75–89	-PN	-	-	72.2;18	-	-	-	-

All codes and abbreviations are the same as in Table 4 except for an added category T for trauma-killed animals (across all sample source categories) in the second column and the added column for PR_mat_ (pregnancy rates of mature females). The latter column also contains information on N_mat_, which is the number of mature females included in the estimation of PR_mat_. Because females without age estimate may be included in N_mat_, this number is sometimes higher than N_R_, which refers to females included in calculations of age at maturity. Curly brackets indicate that the given pregnancy rate is based on a seasonal subset of the sample, which may exclude the perinatal period {-PN} or include only the perinatal period {PN}. Pregnancy rates are generally based on presence/absence of a foetus except were marked with a ^c^ indicating an estimate entirely or partly based on the presence/absence of *a corpus luteum*. A question mark denotes unclear information. Note that sample units 3e and 4a are represented by the same pooled data set for age at maturity.

An alternative analysis (not shown) was run based on the assumption that the unusually long three-year-old female and the old female with missing ovary data, were both sexually mature. The best model for this data set still showed a significant but smaller difference in MAM between Su_1a_ (3.2 years) and Su_1b_ (3.7 years).

Since the model based only on documented reproductive data produced the most conservative A_50_, this model was used to derive estimates of L_50_ by entering A_50_ values from the most parsimonious Richards model into the most parsimonious model for length-at-age. Confidence intervals were estimated by entering maximum and minimum values for both age and length parameters into the length-at-age model. For both age reading practices, L_50_ was about 4 cm larger for Su_1b_ than for Su_1a_ (see Table 5) and estimates based on R1_GLG_ were about 3 cm higher than for R2_Stdg_. Due to very wide confidence limits, none of these differences, however, appear to be statistically significant. Based on R2_Stdg_, L_50_ was estimated at 144.9 (95% CI:128.2–158.7) across sample units.

For comparisons between studies, MAM and A_50_ based on fitted Richards curves were also estimated for data previously analysed in [22, 36, 37], while ASM_SOFI_ for unsmoothed proportions immature was calculated for data from [37, 41]. These estimates are presented with other literature values in Table 6. Generally, estimates of MAM and MAM_SOFI_ were rather similar, but differences of 0.5–1.5 years did occur. Estimates of A_50_ were generally lower than MAM and a difference by up to one year was seen between estimates of A_50_ based on a logistic curve and an unconstrained Richards curve. The latter produced the highest A_50_, but only provided a negligibly better overall fit (ΔAIC = -0.1) than a logistic curve.

Among 24 females with clear signs of previous ovulation (CA, CL or foetus), 22 had a foetus, resulting in an overall pregnancy rate of 0.92 (0.72–0.98, 95%CI). The two barren females were both from Su_1b_ and sampled in the end of March. With a total of 11 mature females, Su_1b_ therefore had a pregnancy rate of 0.82 (0.49–0.96, 95%CI). Including sample unit as an explanatory variable in a binomial GAM did, however, not reduce AIC (ΔAIC_icpt_ = 0.4). Adding the two previously mentioned barren females without evidence of ovarian *corpora* but length >160 cm to the group of barren matures, resulted in an overall pregnancy rate of 0.85 (0.65–0.94, 95%Cl) ranging between 0.93 (0.62–0.99, 95%CI) and 0.75 (0.44–0.92,95% Cl), for Su_1a_ and Su_1b_, respectively. The sample unit effect was, however, not significant (ΔAIC_icpt_ = 0.4). For the later meta-analysis of pregnancy rates, we chose to include the 18-year-old female >160 cm with no foetus and incomplete ovary data among the mature (N = 25) but barren (N = 3) females. This resulted in an overall pregnancy rate of 0.88 (0.68–0.99, 95% CI) (see also Table 6). Both this overall estimate and the separate estimates for Su_1a_ and Su_1b_ were significantly (*p*<0.01 to *p*<0.05) higher than the pregnancy rate of 36.4 (0.14–0.67, 95% CI) estimated for Norwegian harbour porpoises in 1988–90 by [36].

Among nine pregnant females in Su_1b_ (bycaught 7 February -1 April 2017), only two had milk in the mammary glands. These females were bycaught 28 March and 1 April, respectively. Based on the estimated peak birth date on 23 May, the lactation period of these two females appears to be at least 10 months. Only two of 10 females with a CA in spring 2017, were not pregnant.

### Effects of extrinsic factors, COD and sampling areas on pregnancy rates

**Fig 8:**
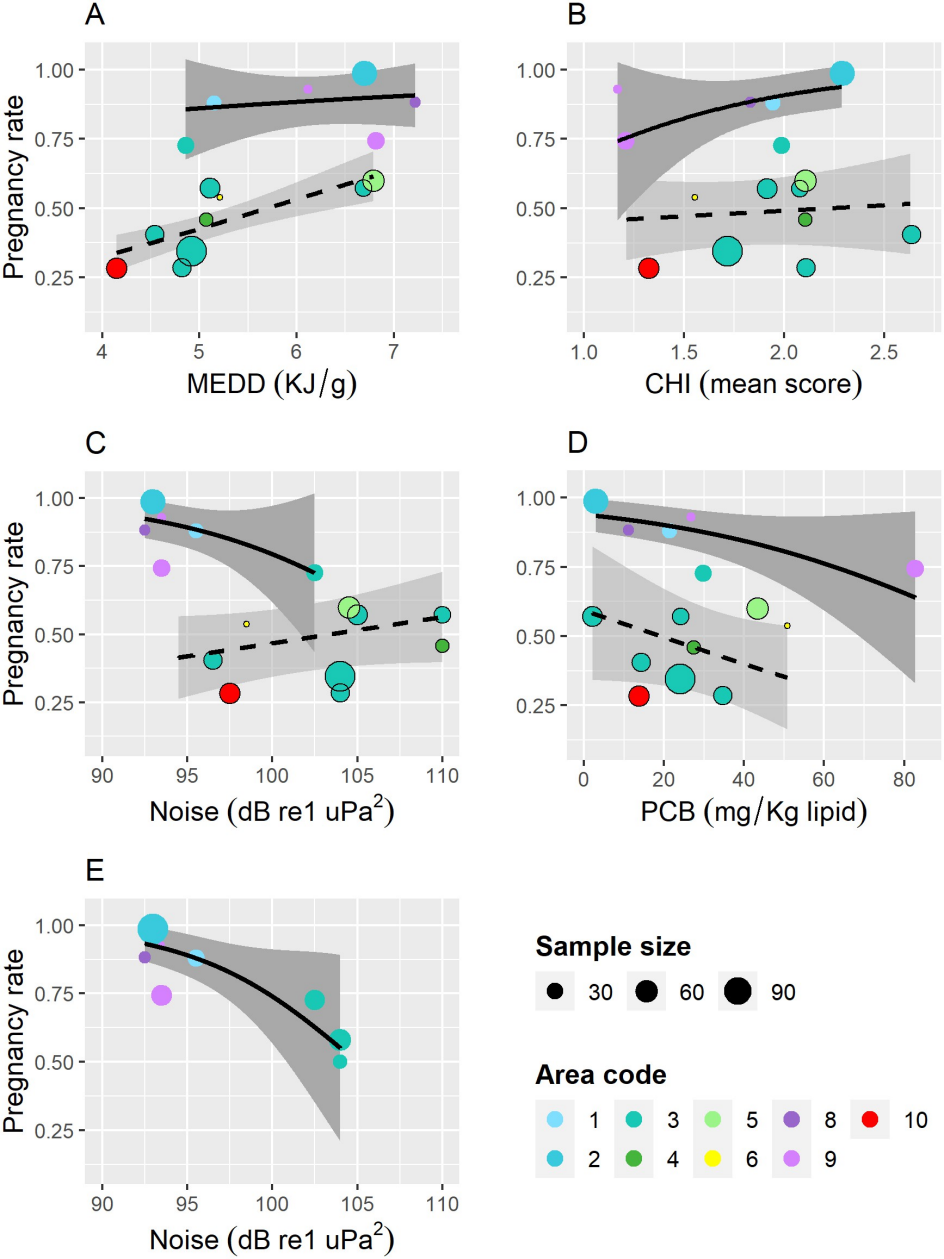
Generalised linear mixed models (GLMMs) for pregnancy rates in trauma-killed (solid lines) and mixed COD samples (dashed lines) in models for (A) MEDD, (B) Noise, (C) CHI and (D) PCB based on 15 data points from nine assessment units/areas (random effect). Symbols show datapoints for trauma-killed (no contour line) and mixed COD samples (black contour lines). (E) Best single predictor GLMM for pregnancy rates in extended data set for trauma-killed samples. Shades show 95% CI.

Effects of the four extrinsic predictor variables (MEDD, PCB, CHI and Noise) and the intrinsic predictor COD were initially investigated in separate mixed effects models for each variable applied to the data set previously used by [17]. The response variables and explanatory variables for this analysis (Meta-Analysis 1) are shown in S2 and S3 Tables, respectively. None of the four extrinsic explanatory variables were significantly intercorrelated (*Pearson*, *p*>0.1) for this analysis. A model with only an intercept term was also run for reference. All models included a random effect term (intercept) for assessment unit (area code). The intercept-only model had an AIC of 115.3. In models with only one predictor, the largest reduction in AIC was seen for COD (ΔAIC_itcpt_ = -18.1). Moderate AIC reductions were seen for MEDD (ΔAIC_itcpt_ = -2.9) and PCB (ΔAIC_itcpt_ = -2.8), while positive changes were seen for CHI (ΔAIC_itcpt_ = 2.0) and Noise (ΔAIC_itcpt_ = 1.9). No configuration of additive effects for the four extrinsic predictor variables produced AICs lower than the model including only COD (AIC = 97.1) but combining extrinsic predictor variables in most cases improved the single predictor models. This was also true for the additive model for Noise and MEDD. Entering COD as an additive term into models for each of the four extrinsic variables reduced AIC substantially in all cases (ΔAIC>-18.7). Entering an interaction term with COD significantly improved AIC for Noise (ΔAIC = -4.0), but not for any of the other extrinsic predictors. This is consistent with the trends observed in models fitted separately to each COD level for the four extrinsic pressures shown in Fig 8A–8D.

The interaction model for COD and Noise showed a significant slope of -0.14 (± 0.06 SE) for the trauma-killed porpoises (*p* = 0.04), but no significant slope for the mixed COD category (*p* = 0.08). The difference in slope for the Noise effect between COD levels was highly significant (*p* = 0.004) but AIC was still significantly higher than for the models for MEDD+COD (ΔAIC = 5.2) and PCB+COD (ΔAIC = 4.2). A stepwise elimination process applied to a model with additive terms for all predictors retained COD, MEDD and PCB in the final model (AIC = 75.6). The positive slope for MEDD (0.64±0.11 SE) and negative slope for PCB (-0-02±0.01 SE) in this model were both highly significant (*p*<0.001).

The alternative analysis based only on trauma-killed samples included two additional data points, not included by [17], i.e., a total of eight data points. This data set is referred to as Meta-Analysis 2 in our S2 and S3 Tables showing the exact values and meta data for the response and explanatory/predictor variables, respectively. Three samples were from the North Sea area, while the rest represented six different assessment units/areas across the North Atlantic (See Fig 8E). An area- based random effect was included to account for potential autocorrelation within the North Sea assessment unit. For this analysis, a significant negative correlation coefficient of -0.88 was found between the MEDD and the Noise variables (*Pearson*, *p*<0.01). AIC for the intercept-only model was 48.2 and no significant reductions in AIC were achieved by single predictor models for MEDD (ΔAIC_itcpt_ = 1.8) or CHI (ΔAIC_itcpt_ = 1.2). A single predictor model for PCB reduced AIC by 2.1 while a single predictor model for Noise reduced AIC by 3.3. The latter model had a significant negative slope of -0.21 ± 0.09SE (*p* = 0.001) (Fig 8E). A substantial improvement was achieved by adding a term for PCB (ΔAIC = -8.0). This model showed highly significant negative slopes for both Noise (= -0.21 ± 0.04 SE, *p*<0.001) and PCB (= -0.03 ± 0.01 SE, *p*<0.001). As evident from Fig 8E, much of the negative relationship between noise and pregnancy rates is driven by low pregnancy rates in the heavily trafficked North Sea area. Entering a binary factor for North Sea origin vs non-North Sea origin to the intercept model reduced AIC by 1.2 units and showed a marginally significant negative coefficient (= -1.84 ± 0.82 SE) for non-North Sea origin (*p* = 0.03). AIC was reduced by -7.4 units by adding a term for PCB.

## Discussion

This study has explored the importance of methodological choices and level of detail in estimation of standard life history parameters of harbour porpoises. The main focus was on recent data from Norwegian waters, but compilations of similar data from the entire Atlantic region suggest that our findings are relevant for the comparability between many studies and therefore for overarching analyses of the factors affecting life history parameters of this species. The importance of methodological homogeneity at the initial sampling level was clearly shown by the highly significant effect of cause of death in our meta-analysis of factors affecting adult pregnancy rates.

### Comparison of age estimation practices

Our analyses demonstrated the potential significance of early GLG completion for age estimation of young porpoises. R2_Stdg_ estimates were not significantly different from pure GLG counts (R1_GLG_) in autumn samples but were one year lower than R1_GLG_ in two-thirds of the spring samples. In these cases, the R2_Stdg_ reading practice assumes that the last boundary layer was formed within the last few weeks or months, thus representing the age in the upcoming calving season. This is similar to reports from Icelandic waters by [41], who chose to exclude such GLGs from age at maturity analyses to avoid mixing up age at ovulation and age at parturition. The general lack of attention to this phenomenon in existing age reading protocols may suggest that it is less common in temperate areas which were the primary centre of research prior to the age reading workshop held by the International Whaling Commission in 1990 [30]. One influential study from 1972 based on Danish harbour porpoises [38], stated that GLGs were completed right around the calving season in summer, which would ensure almost perfect alignment of integer age and the number of GLGs. Another study from the Bay of Fundy [39] reported some cases of GLG completion a few months prior to the peak calving period, but the prevalence was not stated.

Little is known about the biological mechanisms that control the timing of boundary layer formation in porpoises and other odontocetes [31, 61]. Generally, however, boundary layer formation seems to coincide with periods of nutritional or energetic stress [34, 65]. In Norwegian waters, late winter/early spring indeed seems likely to be the most energetically stressful period, since water temperatures are at their annual low and prey species are likely at their lowest energy density after a long dark winter with no primary production [84, 85]. This also appears to be the time of weaning of harbour porpoises in Northern waters based on our own limited data and Icelandic data presented by [41]. The reported boundary layer formation during summer in Danish waters also appears to coincide with the reported time of weaning in the North Sea area [17]. Summer may generally be a period of reduced primary production in the North Sea area, since the main peak in phytoplankton blooms has been reported to occur in spring and autumn [85]. The latter may, however, be of less consequence to harbour porpoises in shallow waters due to easy access to benthic prey. Water temperatures in the Southern North Sea are also approaching their annual high during this period [86], which may reduce energetic stress. On the other hand, summer is the period of energetically demanding activities like mating, birth and early lactation [17] and possibly moulting of skin [87], which may all impose nutritional and/or energetic stress. Overall, the existing data therefore may be consistent with a link between energetic stress and boundary layer formation.

In nine of the fourteen studies of North Atlantic harbour porpoises listed in Tables 4 and 6, the stated age assignment criterion in analyses of age at maturity is the number of complete GLGs. Of the remaining five studies, two have attempted to estimate what we have termed “standard age” (referring to the most recent calving season), while the other three have either used decimal GLG counts (GLG+) based on the relative thickness of post boundary layer dentine or used this feature to estimate integer age in the nearest (future or past) calving season (GLG++). The first approach is likely to cause a progressive increase in estimated age at maturity for samples collected over the course of the gestation period. The latter will cause a sudden shift about halfway through the gestation period, which is very similar to the effect of early GLG completion. This diversity of seasonal reference points is likely to introduce noise and biases in many age-related life history parameters. Increased efforts to standardise seasonal reference points for age estimates therefore seem warranted.

As individuals age, the width of the GLGs decreases, making it more challenging to assess the relative thickness of the dentine layer formed since the last boundary layer. In many cases, the boundary layer gradually becomes the widest layer in the GLGs [30]. A deviation of -1 year was still the most commonly occurring difference between R2_Stdg_ and R1_GLG,_ for porpoises with 4–5 GLGs. From age four, however, between-reader differences become more pronounced and involve more discrepancies in the counts of full GLGs. Some authors have inferred that considerable underestimation may be expected after the age of seven years [33, 38], which is close to the upper age limit in some published studies (see Tables 4 and 6), and for the R1_GLG_ reading practice in the present study. Similar problems are known from dentine-based age readings in other species [34, 35]. For ringed seals, using cementum GLGs increased the maximum age estimate by 14 years compared to ages based only on GLGs in the dentine [35]. Cementum GLGs are deposited on the outside of the tooth and are less constrained by surrounding structures than dentinal GLGs deposited towards a gradually closing pulp cavity. Including information from cementum GLGs has been recommended to reduce underestimation in older harbour porpoises [32], but no guidance has been given on the best reading positions and all published reference images focus only on dentine [30, 31]. During the present study, we discovered that cementum was deposited very unevenly around harbour porpoise teeth, which may have deterred some readers from using cementum GLGs for age estimation. Based on thorough selection of reading position, cementum GLGs may, however, provide a useful supplement to dentinal GLGs.

The maximum ages previously reported for harbour porpoises in Norwegian waters (Tables 4 and 6), as well as the R1_GLG_ estimates for females in the present study, are markedly lower than the maximum ages reported in most other studies (Tables 4 and 6). Similar maximum ages have only been reported from the Swedish Skagerrak coast (analysed in the same study as the older Norwegian data) and Newfoundland. The reason for this is unclear, but details of GLG structure and readability have been found to differ between geographical areas [31, 37, 75]. In the present study, ages based on the R2_Stdg_ age reading practice were sometimes substantially higher than for R1_GLG_. This was often due to greater emphasis placed on maximum GLG counts in the root dentine or in the cementum as exemplified by our S1 Fig. The higher estimates were generally more consistent with biological data, as outlined in the results section. Comparisons of dentine-based age estimates with estimates based on aspartic acid racemization analysis have also shown examples of up to nine years higher estimates based on the latter method [88]. More thorough studies are therefore needed to validate and guide the relative weighting of GLG counts in various tissues and reading positions in harbour porpoises.

### Sex ratio/male proportion

Although the postnatal male proportion in the present study was not significantly different from 0.5, the nominal sex ratio of 1.3:1 is consistent with a generally observed pattern of male bias in North Atlantic harbour porpoises reported by [15]. More pronounced and statistically significant examples of male bias have been reported for harbour porpoise bycatches in Iceland [41] and Newfoundland [37]. So far, no significant deviations from an even sex ratio in foetuses have been found in the few available data sets, including that of [41]. Thus, the reported male bias in harbour porpoise populations appears to arise postnatally, possibly due to gender differences in susceptibility to bycatch caused by differences in prey preference, habitat use and/or behaviour [15].

### Dates of conception and birth

The estimated peak date of conception of harbour porpoises in Norwegian waters (1 July) is about one month earlier than for harbour porpoises in the North and Baltic Seas [40], but close to the value for the Bay of Fundy (6 July). Assuming an active gestation period of 10.4–10.7 months [40] and a preimplantation period of 5-weeks, peak birth is estimated to occur in late May/early June in Northern Norway and Bay of Fundy, respectively. This is approximately a couple of months before the sea surface temperature maximum in August-September [84]. Reducing the risk of hypothermia in the new-born calves thus does not seem to be the primary driver of calving phenology in these areas [40, 89]. In populations feeding on pelagic prey over a wide depth range, such as in the Bay of Fundy [90, 91] and off Northern Norway [92, 93], seasonal changes in the vertical distribution of prey could affect the depth and duration of dives and the associated risk of mother-calf separation. Since many pelagic fish feed more in the surface layers during spring and summer, this could select for earlier calving in deep water habitats compared to shallow areas of the North Sea [94], where more benthic diets are observed [17, 92]. Advantages of completing the lactation period before the energetically most stressful period, presumed to occur in late winter, may also have selected for early calving in Northern Norway.

### Postnatal growth

Like all previous studies, we found females to be significantly longer and heavier than males after the early calf stage [17, 19, 60]. Stage-based mean sizes and length-at-age models based on R2_Stdg_ also showed a significant increase in size between sample units of the Norwegian focal data set consistent with a seasonal effect. This supports the relevance of using continuous age assignments for growth analyses of samples collected in different seasons as recommended by [32]. Using the explicitly continuous estimates, R2_Decg_ removed a significant sample unit effect observed in GAMs for length-at-age based on R2_Stdg_. There was also no significant sample unit effect for R1_GLG,_ presumably due to the upward-rounding effect of early boundary layer formation in the spring samples. Both sexes, however, showed clear evidence of sample unit effects within both readings, also when a seasonal correction factor was added to the zero-year age class of the R1_GLG_ readings. This seems mainly due to effects of the calf data, as the mean length of the non-calf stages were highly homogeneous between sample units, especially for the mature stage of both males and females.

Some authors have suggested that the youngest stages of harbour porpoises follow a different growth curve than older age classes, but the estimated cut-off age has varied between studies [95]. This hypothesis is also supported by the present analyses of the focal Norwegian data set, since all the estimated lengths at age zero (l_0_) are substantially larger than the observed lengths of neonate harbour porpoises in the North Atlantic reported to be between 60 and 80 cm [16]. Individual growth curves for five captive harbour porpoises (four females and one male) reported by [96] show accelerated growth during early lactation and a period of reduced growth around the expected time of weaning followed by a new rapid acceleration after about one year of age [96]. Based on this pattern, a higher length at l_0_ and lower growth rate at l_0_ (k_0_) would be expected for autumn samples than for spring samples. This was in fact observed for males in the present study, while the opposite was observed for females. Larger size at birth for males than for females could explain this pattern, but to our knowledge, effects of sex on neonatal size have not been subject to any thorough statistical analyses for harbour porpoises [16, 40, 97]. A one-stage von Bertalanffy growth curve was found to give a significantly better fit to individual growth data for captive porpoises than a one-stage Gompertz curve [96]. Comparisons of one-stage Gompertz and von Bertalanffy growth curves for larger cross-sectional data sets, including the present study, have, however, found slightly better fits for the Gompertz model [37].

Very few studies have fitted two-stage growth models to length-at-age data for porpoises and none of these are included in Tables 4 and 6. In the present study, sample sizes were simply too small to meaningfully explore two-stage growth models and we therefore focused on the more commonly used one stage growth models. The significance of the sample unit effect after seasonal correction of the R1_GLG_ ages for age class zero is, however, an important result, considering the variability in seasonal timing of sampling among existing studies shown in Tables 4 and 6. For Southwestern and Southeastern UK data [20], reported that seasonal corrections for age class zero were carried out for the later data sets (Su_3h_ and Su_5b_), but not for the early data sets (Su_3g_ and Su_5a_). Potential effects of this were, however, not explored. The l_0_ parameter of the Gompertz curve is closely correlated with the length at the point of inflection, which is fixed at 36.8% of L_∞_ [98]. This likely explains the consistent patterns of covariation between the Gompertz parameters in the focal Norwegian data set. When sample sizes are largest in the lower end of the age spectrum, the lengths at age of these age classes will have strong influence on the other two parameters as shown most clearly in the unrealistically high L_∞_ values for females in the unconstrained models for females based on R1_GLG_. These values are selected by the estimation procedure to optimize the shape of the curve for the age range with data and are not penalised simply because there are no data available for the age range when the estimated asymptotic length would be reached. The large confidence intervals around the L_∞_ values in the present study clearly signal the uncertainty of these estimates, but confidence intervals are not always available for older studies as shown in Tables 4 and 6 or taken explicitly into account in verbal comparisons [41, 97, present study]. Not surprisingly, the confidence intervals of L_∞_ are largest for the samples with lowest maximum age such as the R1_GLG_ based results for Norwegian males from Su_1a_ and Norwegian females from both sample units. In the most parsimonious models, the reader effects on L_∞_ were, however, neutralised, due to the imposed constraints. Seasonal correction of R1_GLG_ estimates removed all significant differences between readings and sample units for males, but a significant difference in k_0_ was retained for females. This makes sense as the substantially shorter age span of the R1_GLG_ ages implies a faster increase between the uniform estimates of l_0_ and L_∞_.

In most studies only one age reading practice is used. This increases the risk for methodologically driven differences among studies also for the most commonly compared growth parameter, asymptotic length L_∞_. Taking maximum reported age and indications of seasonality into account may, however, be a useful first step to reduce the risk of methodological bias. Maximum age in the studies shown in Tables 4 and 6 appeared to be somewhat bimodal with most studies showing values close to our R2_Stdg_ based estimates and a smaller group with lower maximum ages. For the first group of studies, *L*_*∞*_ of both male and female North Norwegian harbour porpoises were in the upper range of the North Atlantic estimates outside the range of the large Iberian subspecies of harbour porpoises [63], which is thought to reach into the Celtic Sea area [5, 19] (termed SW.UK in Tables 4 and 6).

Estimates of *L*_*∞*_ from Iceland, Scotland, southern Norway and the Swedish Skagerrak coast were similar to the most parsimonious estimates for the focal Norwegian data set (Tables 2 and 3), while estimates from the Northwest Atlantic and the southern North Sea appeared to be somewhat lower. Most of the asymptotic length estimates for the first group of studies were based on trauma-killed samples, although the Scottish data set was mainly based on stranded porpoises [18]. If old age classes are sufficiently represented to dominate the estimation of asymptotic length, this parameter is not particularly likely to be affected by poorer health in stranded samples. This is because many old animals will have completed their length growth before becoming ill and stranding. Danish wild-ranging porpoises, on average appear to reach 95% of asymptotic lengths at age 3.9 years in males and 4.9 years in females based on data sets comprising animals of up to 18–20 years of age [97].

The samples with large *L*_*∞*_ in Tables 4 and 6 are all from waters of some depth (see Fig 1). Low *L*_*∞*_ is, however, also observed for the deep diving West Greenland porpoises [16, 96], which are thought to constitute a distinct cold adapted ecotype with a higher weight/length ratio [6, 7] than neighbouring populations. Changes in asymptotic length over time have been suggested for females in the Bay of Fundy area [63] but appear questionable due to large confidence intervals around the presented estimates of *L*_*∞*_. No changes in *L*_*∞*_ are seen over time for Southwest and Southeast UK waters based on samples with high upper age ranges [19].

Not surprisingly, morphological features associated with dive capacity and prey capture seem to be under selection in harbour porpoises [4, 65, 99–101]. Differentiation in skull morphology within Danish waters are thought to represent specialization to benthic versus pelagic feeding, which appears to be a defining feature of harbour porpoise biology (see also [100, 101]). It therefore seems plausible that differences in *L*_*∞*_ between samples from the shallow southern North Sea and the deeper areas of the North Atlantic could be at least partially driven by long term natural selection, although no clear genetic factor has been identified. Recent genetic analyses have, however, suggested selection pressures driven by salinity gradients [3], which are likely also correlated with depth gradients since fresh-water inputs are generally from land within the main distribution area of harbour porpoises.

### Male reproduction

No significant effects of age reading practice or sample units were seen for male age at maturity estimates for the focal Norwegian data set. This may, however, be due to low statistical power resulting from low sample sizes, as estimates differed by 0.5–0.6 years between R1_GLG_ and R2_Stdg_ for Su_1b_ based on CTW_100g_ (Table 4). The higher estimates for R1_GLG_ ages were mainly due to an extra year assigned to six immature males with a recently formed GLG boundary layer. None of the males with R2_Stdg_ = 1 year had a CTW>100g in spring. Interestingly, most of the males with R2_Stdg_ = 2 years in the autumn samples did have CTW>100g suggesting that our spring samples may have been collected too early to capture the maturation of the youngest males. Both seasonal dynamics of CTW and age reading approach may therefore be expected to affect estimates of male age at maturity.

Estimates of A_50_ were generally lower than MAM estimates (up to 0.9 years), particularly if the underlying model was allowed to differ from a logistic curve (Table 4) which provided a significantly poorer fit to maturity curves based on CTW_100g_ in our study. These two estimates of age at maturity should therefore not be considered directly comparable. MAM and ASM_SOFI_ estimates should provide identical estimates if based on perfect mirror images of age-specific proportions mature/immature. Differences may, however, arise if one estimate is based on smoothed proportions and the other is not. The latter is often the case for ASM_SOFI_ estimates in the literature such as [19]. Hence, the MAM value estimated by us for bycaught UK males based on data reported by [19] was 0.8 years lower than the ASM_SOFI_ based on raw proportions for the same data (see Table 4, Sample unit 3gh+5ab). This is likely due to a highly atypical increase in proportions immature by 72% between age two and three, evident in the supplementary data file of [19].

Overall, estimates of male MAM and A_50_ based on CTW_100g_ in the present study were slightly below previous estimates for the North Atlantic (Table 4). The values closest to ours are from other studies based on trauma-killed animals in Iceland and West Greenland (Table 4). These are on average expected to be more fit than stranded animals and therefore also likely to mature faster [18, 19]. MAM for male porpoises from Newfoundland and the Gulf of Maine were somewhat higher than for Norwegian males (Table 4). The Newfoundland sample was collected almost exclusively during the assumed mating period in July and the maturity criterion was presence of secondary spermatocytes, spermatids or spermatozoa [37]. No males below the age of three years fit this criterion even if several two-year-olds had CTW>100g and even >200g [37]. A vigorous increase in CTW is observed around the mating period and the CTW-at-age relation in [37] is therefore not expected to resemble that in our study or in [41]. The low age at maturity estimates for West Greenland estimated by [52] were also based on samples collected within the breeding season. Unlike [37], however [52], accepted spermatogonia as a sign of maturity, which likely includes more of the youngest males and reduces age at maturity estimates.

The highest reported A_50_ value of five years for male harbour porpoises (Table 4) is based on stranded samples collected around Scotland throughout the year [18]. This study used several histological criteria for maturity including the average diameter of seminiferous tubules (ADST) with a stated typical value for mature males of 200 um. This is in the absolute upper range of ADST reported in other studies [41–43] even during the peak breeding season. This very conservative criterion could therefore have contributed to an unusually high A_50_. The ADST criterion used for Icelandic porpoises was similar to previous studies for the North Atlantic [41–43]. Less than 1% of the males with CTW>100g was classified as immature in [41] and the misclassification rate seemed to increase for higher CTW criteria. We therefore believe that a CTW criterion of 200g would be misleading for our data set and likely all data sets sampled well out of the breeding season. Since CTW_200g_ has previously been suggested as a universal maturity criterion for harbour porpoises [75], we did, however, provide supplementary estimates of MAM and A_50_ based on this criterion. This increased both MAM and A_50_ by one year and would change the biological interpretation of male reproductive parameters for our area and across regions. The complexity of histological maturity classification for males collected outside the breeding season therefore appears to increase the risk of methodologically induced differences between studies. Including calculations based on a more easily transferrable criterion like CTW may therefore be advisable. The chosen threshold value must, however, take seasonal dynamics into account.

The estimated length at 50% maturity based on CTW_100g_ was slightly smaller for growth curves based on R2_stdg_ ages than for R1_GLG_ ages due to the lower length at birth estimated for the former. Lengths at 50% maturity estimated for both age reading practices were in the lowest range of previous studies (Table 5), although our estimated male asymptotic lengths were in the highest reported range (Table 4). Early onset of maturity in North Norwegian male porpoises therefore does not appear to severely limit continued somatic growth.

### Female reproduction

#### Patterns of corpora accumulation with age

Although the focal Norwegian data set for mature females was too small to fully investigate effects of both age reading and CA classification practices on the *corpora* accumulation pattern, several results and observations suggest that both play a role. For example, a significant change in slope of the age-specific *corpora* counts due to CA classification practice was only seen for R2_Stdg,_ and the estimated age-specific slope of *corpora* accumulation was up to twice as high for R1_GLG_ as for R2_Stdg._ Even the highest estimated slope of the most parsimonious Norwegian models was, however, only around 0.5 *corpora*/year and hence did not support annual formation of one persistent *corpus*. This pattern was both due to excess CAs in young females and lower than expected numbers of CAs in older females. The latter was most pronounced for R2_Stdg_ estimates. Excess CAs in young females were most common for R1_GLG_ estimates and involved both interior and surface CAs for this age reading practice.

For the focal Norwegian data set, the individual maximum numbers of excess CAs were estimated at one CA for R2_Stdg_ ages and 3–4 CAs for R1_GLG_ ages. These numbers are similar to results that can be inferred for West Greenland [52], whereas German data from [61] show up to five excess CAs and corpora counts in [41, 45] suggest up to nine and 10 excess CAs, respectively. Sample sizes likely affect the probability of observing extreme values, but the very occurrence of large numbers of excess CAs and the differences between studies based on large samples like [41, 45, 61], do support the hypothesis, that there are considerable potential uncertainties in the interpretation of structures used for determination of reproductive status and/or age of female harbour porpoises. Excess *corpora* have so far mainly been explained as regressing *corpora lutea* from poly-ovulations during the first breeding cycle(s) [45, 47, 50, 51], but the large numbers in some studies suggest that some of them could also be *corpora atretica a* as reported for pilot whales [55] and also suggested for harbour porpoises by [102]. Several other types of atretic follicles have also been described for pilot whales [55], but they appear to be more macroscopically distinct from CAs than *corpora atretica a*. Underestimation of age may also inflate the perceived number of excess CAs as shown by the differences between the two age reading practices for the focal Norwegian data set. Theoretically, a few occurrences of excess CAs could also be due to simultaneous double ovulations. The double CLs recorded in two out of 39 females by [61] suggest that traces of one double ovulation is not unrealistic among the 19 mature females with complete ovary records in the focal Norwegian data set. Twinning rates in cetaceans are generally reported to be very low [28] and fatal to the mother if taken to term. One case of twin foetuses has, however, been reported for harbour porpoises [103] as well as one set of neonate conjoined harbour porpoise twins [104]. These cases would most likely have resulted in the formation of two genuine CAs during one reproductive cycle.

For both R1_GLG_ and R2_Stdg_ ages, the age-specific accumulation patterns for *corpora* counts based on interior and total CAs were statistically indistinguishable from the included German and West Greenland data sets, respectively. The similarity of the data from West Greenland with the focal Norwegian data for total *corpora* counts could be related to the participation of a common ovary reader in both studies. Both the West Greenland study and the German study aimed to exclude atretic follicles from their *corpora* counts but based their CA classification on two different protocols ([29, 54], respectively). Unlike most other studies of harbour porpoises, the German study [61] also used histological staining (with Massons trichrome) to identify connective tissue as a basis for identifying CAs. The exact histological criteria for this procedure are, however, not stated. Previous studies of common dolphins (*Delphinus delphi*s) have suggested that the relative content of elastin in small CAs may be used as a guide to whether a given *corpus* resulted from a pregnancy or an infertile ovulation [49]. No simple presence/absence based histological criterion has, however, to our knowledge, been devised to distinguish between *corpora atretica a*, *corpora* from infertile ovulations and CAs resulting from active gestation. All of these structures have, however, been assumed to regress towards the surface [55, 61], and are in the final stages likely to contain small absolute amounts of connective tissue. This could lead to frequent exclusion of surface structures and hence explain the similarity between the accumulation pattern based on the German data and the Norwegian interior *corpora* counts. The German data set is, however, also characterised by very low *corpora* counts in many females older than five years compared to most other data sets [37, 41, 47, 52], including the focal Norwegian data set for interior *corpora*. This might suggest effects of other factors such as general health or technical quality of the samples, which could all be related to the fact that the German data set was mainly based on strandings, while the other samples were all from trauma-killed animals. High *corpora* counts have, however, also been reported for stranded porpoises from the United Kingdom [45]. These were explicitly reported to be based on structures seen in the ovary surface and could therefore be partly comprised by structures fitting the description of *corpora atretica a* given by [55].

The high back-calculated ages at maturity found for the Norwegian data based on the R2_Stdg_ age reading practice, support the hypothesis that not only *corpora atretica*, but also *corpora* of ovulation and pregnancy may be resorbed during the female’s lifetime as suggested by [29, 37, 55, 61]. Rejection of pure surface CAs in older animals may therefore lead to misclassification of maturity status. In the focal Norwegian data set, surface CAs always occurred together with a CL, but this may not be the case in samples collected between reproductive cycles or from populations with low pregnancy rates. The *corpora*-based pregnancy rates for the German samples included in the meta-analysis by [17] and the present study (see S2 Table) are in fact rather low (0.46–0.57) compared to pregnancy rates for several other data sets including the focal Norwegian data set (0.88). For the German data set, several older females (5–14 years) without any *corpora* appear to have been included in calculations of age at maturity in [61] resulting in an unusually high A_50_ of five years (Table 6). It cannot be excluded that a more inclusive CA classification practice might have resulted in a positive maturity status for some of these individuals.

In contrast to the present study and most other published studies [41], found an age-specific *corpora* accumulation rate of 0.98 corpora per year consistent with lifelong persistence of *corpora* from annual ovulations. This study was based on bycaught Icelandic animals and explicitly referred to the pilot whale protocol in [55] for exclusion of *corpora atretica* and appears to show a very low rate of excess CAs among young and middle-aged females. One case of a one-year-old female with one *corpus* could be due to seasonal age estimation problems. A few conspicuous cases of many excess CAs in females older than eight years could be due to underestimation of age.

The very high maximum number of non-excess *corpora* found in older females by [41] suggests a higher CA detection rate than in most other studies. This could be due to the use of a ten times higher magnification (X40) than for example [61]. Most other studies do not specify magnification (e.g. [18, 19, 52, 65]) and some studies are explicitly based entirely on naked eye observations [17, present study]. The minimum reported CA diameter of about 2mm by [41] should also be visible under lower magnification and even to the naked eye. The features defining a CA under high magnification may, however, differ from those at lower resolution. Details of sample preservation, such as decomposition and/or freezing prior to fixation may also affect the visibility and distinctiveness of ovarian *corpora* [44]. More direct comparisons of all the mentioned methodological factors are needed to assess the comparability of *corpora* accumulation patterns between studies and their relationship with external factors such as contaminants, disturbances and diets. Based on available evidence it cannot be excluded, that differences in *corpora* classification practices may in some cases also affect estimates of pregnancy rates and female age at maturity.

#### Age and length at maturity

For the Norwegian focal data set, female MAM and A_50_ were 0.5–1.0 years higher for Su_1b_ than for Su_1a_. This difference was only independently significant for the R1GLG—based estimates (~1 year), but an overall increase in MAM and A_50_ was retained in the final joint model for the two readings. This difference between sample units is consistent with expected seasonal effects of early GLG completion on R1_GLG_. The fact that there was a similar although less pronounced pattern in the R2_Stdg_ based estimates may suggest an additional spatiotemporal effect, but larger and less confounded samples are required to resolve this. Su_1a_ had a more southern centre of gravity in Northern Norway and comprised a few samples from the Norwegian North Sea area. The same is true for the Norwegian data from 1988–1990 analysed by [36]. Estimates of MAM, ASM_SOFI_ and A_50_ for the latter sample were all close to four years and thus closest to the sample from the northernmost recent sample. Unlike both recent samples, however, the older sample was primarily collected during late spring and summer. Since age estimates in [36] were based on full GLG counts, many of these females were likely assigned the age attained in the upcoming calving season rather than during the most recent ovulation period. A positive bias is therefore generally expected compared to the recent samples, particularly those based on R2_Stdg_ which explicitly refers to the age at last ovulation. Whether or not this type of seasonal bias also occurs for samples from other areas depends on the timing of sampling in relation to boundary layer formation, which is generally not explicitly reported. Extensive pooling of data across seasons in many studies (see Table 6) does, however, suggest that seasonal biases may potentially affect the accuracy and comparability of female age-related life history parameters.

As for males, differences in mathematical formulas used to calculate female age at maturity were found to generate considerable differences within and between studies. Most notably, the estimated A_50_ for Norwegian samples from 1988–90 increased by one year when allowing the Richards function to take other shapes than the logistic curve (Table 6). This data set was, also notable by comprising mature females among the age class of one-year-olds, possibly reflecting the previously mentioned seasonal age estimation problems.

#### Pregnancy rate

Since abortions are reported to be common among harbour porpoises in some areas [19, 22], estimated foetus-based pregnancy rates are likely to decline over the course of the gestation period [53]. In the focal Norwegian data set, the nominal pregnancy rate was indeed lowest in the spring sample, but sample sizes were too small to show a significant difference between the two spatiotemporal sample units. Estimates of adult pregnancy rates would also be sensitive to any difficulties in identifying signs of previous reproductive cycles in females that are not pregnant at the time of sampling. In our study, it is for example noticeable that four out of five three- year-old females bycaught in autumn (13 September- 10 October) were pregnant, while none of the three three-year-old females caught in spring (7 Feb-3 April) showed any signs of sexual maturity, even though one of them was 161 cm long. In a study from the Salish Sea, all harbour porpoise females >155 cm were considered sexually mature and included in calculations of pregnancy rates [78]. Applying the same criterion to Norwegian data reduced the overall pregnancy rate from 0.88 (±0.15, 95% CI) to 0.84.6 (±0.16, 95% CI), but it is still one of the highest pregnancy rates reported in the North Atlantic (Table 6). Different practices for including large or old females without ovarian corpora among the mature but barren females may, however, lead to significant systematic differences between studies.

As described earlier, available evidence on persistence of *corpora* from infertile ovulations is very uncertain in harbour porpoises, especially for first time ovulators, if they are not successfully mated and fertilised. Regardless of the exact selection of maturity criteria or sample units, the estimated pregnancy rates for the focal Norwegian data set were, however, at least twice as high as reported for Norwegian harbour porpoises for the period 1988–90 [36]. For the latter data set, the estimated early pregnancy rate (~ovulation rate) based only on CLs was almost identical to the foetus-based pregnancy rate [36]. Since all mature females in this previous data set were sampled in May-July, the low estimates of pregnancy and ovulation rates are likely due to a high prevalence of females sampled between parturition and ovulation. In comparison, the partly or fully *corpora*-based pregnancy rates estimated for the Bay of Fundy and Eastern Newfoundland based on samples collected in July and August were both similar to foetus-based estimates for the Northwest Atlantic. For the Northeast Atlantic in general, the most conspicuous differences in pregnancy rates appeared to occur between stranded and bycaught samples suggesting a link to cause of death (Table 6). High incidence of females in poor health among stranded samples is also evoked by [78] to explain the very low pregnancy rate estimated for the Salish Sea in the Northeast Pacific.

### Effects of extrinsic factors, COD and sampling areas on pregnancy rates

Female reproductive rates are widely considered to be important determinants of population growth rates of harbour porpoises [20, 21, 61]. In most management regions, including Norwegian waters, the spatiotemporal resolution of reproductive data is, however, lower than the spatiotemporal variability in pressures which may affect reproductive rates [7, 8, 14, 15]. Meta-studies like the one undertaken for female age at maturity and pregnancy rates by [17] may therefore potentially provide important general information on expected effects of various pressures. The results are, however, sensitive to the choice of predictors and to methodological differences between studies in the estimation of response variables.

The present study has highlighted the extensive amount of methodological heterogeneity in published estimates of female age at maturity in harbour porpoises, which may at least partly explain why [17] did not find any clear patterns in their meta-analysis of extrinsic effects on age at maturity estimates. In contrast, their meta-analysis of the methodologically simpler data set of pregnancy rates did show a clear positive correlation between pregnancy rates and the estimated mean density of prey (MEDD) and to some extent also showed an effect of PCB levels. Our extended analysis of the pregnancy rate data set studied by [17] identified COD category as a much stronger predictor than any of the previously tested predictors. Effects of MEDD and PCB, were, however, still significant when added to models also including COD. In a local analysis of Dutch harbour porpoises [17], did identify overall health status as the most important predictor of pregnancy status, but the authors did not show a direct link with COD category and did not include COD category as a factor in their global meta-analysis of pregnancy rates. A smaller scale meta-study for southern UK waters [19] did not find any statistically significant effect of COD category (trauma-killed versus infectious diseases) on pregnancy rate and age or length at maturity.

In our rerun of the meta-analysis of [17], MEDD was the only extrinsic predictor variable which had a significant effect on pregnancy rates when entered alone for the full data set. MEDD was also found to be the most influential extrinsic parameter in the original study, but the authors voiced some concern that inclusion of stranded animals in poor health might have confounded the apparent effect of MEDD. This was based on the rationale that poor health may independently reduce both the likelihood of becoming pregnant and the ability to capture prey of high energy density. The latter are typically fast-swimming pelagic fish [17], which are likely harder to catch than other prey, particularly for animals in poor health. Our study supports this concern, as we did not find any significant effect of MEDD on pregnancy rates in analyses based only on trauma-killed females. It seems biologically reasonable that MEDD could play a role in determining pregnancy rates, but the limited diet data available for this analysis does not show it.

In our analysis based only on trauma-killed samples, vessel noise (Noise) was the only predictor variable with a significant effect, when entered alone. Harbour porpoises are disturbed by vessel noise [80] and it seems plausible that this could reduce feeding efficiency and the resources available for reproduction. It is, however, notable that the negative relationship between pregnancy rates and vessel noise is to a large extent driven by very low pregnancy rates from heavily trafficked areas in the southern North Sea. Several life history characteristics of harbour porpoises from this area appear to differ from populations in more oceanic parts of the North Atlantic, including timing of births in relation to water temperature and primary productivity. Being born in summer is likely thermodynamically advantageous to the calf. On the other hand, this timing of births means that parturient females in the North Sea must rebuild resources for the upcoming breeding season after the spring bloom when energy-rich pelagic fish are likely less concentrated in the upper layers of deeper waters. There is also evidence that lactating harbour porpoise females in the North Sea tend to inhabit very shallow waters [94], where diets are generally dominated by less energy rich prey benthic prey [17, 93, 98].

A large reduction in female blubber thickness has been documented in females from the southern North Sea during the period corresponding with early lactation [17]. Throughout the year, nutritional status in samples from this area is furthermore positively correlated with the likelihood of ongoing pregnancy and size of the foetus [17]. These relationships make biological sense, but the apparent preference for shallow waters by lactating females [94] also suggests a possible confounding effect between lactation status and feeding on benthic prey in the first place. The added energetic demand of ongoing lactation also seems likely to be associated with overall lower nutritional status and thereby reduced likelihood of a successive pregnancy or reduced foetal size compared to non-lactating females. No considerations of these potential effects of lactation status are mentioned in [17]. A comprehensive study of health and reproductive status of female harbour porpoises in all COD categories from U.K. waters [22] found that lactating females (N = 6) were generally in better health than other reproductive categories, but none of them appeared to have an active pregnancy of the new cycle. Failure to recover fast enough to become pregnant again shortly after a successful birth with ensuing lactation is presumably not uncommon given the rather short time window between reproductive cycles in harbour porpoises. High lactation rates and/or any sampling selectivity towards lactating females may therefore affect both the actual and estimated pregnancy rates. Whether or not skipping the next pregnancy is an overall detriment to the population growth rate, however, depends on potential gains in terms of calf survival.

In more oceanic areas like Northern Norway, Iceland and North America, there seems to be a better match between the post-parturient recovery period and the seasonal access to high energy prey. Colder water temperatures furthermore favour food chains based on more lipid-rich zooplankton species like *Calanus finnmarchicus*, compared to the southern North Sea [105]. These factors could partly explain the higher pregnancy rates in harbour porpoise populations from these colder and deeper waters. For the overall reproductive success, some of these advantages could, however, be offset by potentially less favourable postnatal conditions for the calf due to lower water temperatures and increased risks of mother-calf separation because mothers must likely dive deeper for prey during winter. Calves living in deep water habitats also have less opportunity to supplement their food intake with easily caught shallow water prey. Independent feeding has been recorded in calves during their first winter in both Scotland [18], the Bay of Fundy [106] and for the focal Norwegian data set [93], but the associated risks for mother-calf separation are unknown. Preweaning calf loss likely increases a females chance of fast recovery between reproductive cycles and hence the chance of successive pregnancies [28].

The mentioned caveats suggest that high pregnancy rates do not necessarily translate into a higher population growth rate. Low estimates of age at maturity and high somatic growth rates may also be deceptive in this respect. This paradox could explain why, the southern North Sea, despite record-low pregnancy rates, has had the largest concentration of harbour porpoises over the last 20 years with no sign of a general decline [107]. In comparison, the density of harbour porpoises in Norwegian waters is rather low [7, 14, 107]. For the focal Norwegian data set, lactation status was only available for spring samples and therefore could not reveal any premature weaning. With only two lactating females, this data set was also too small to provide reliable inference on the likelihood of successive successful pregnancies. More systematic studies of the seasonal occurrence of lactating parous and/or pregnant females may shed more light on the effects of lactation duration and preweaning calf loss on pregnancy rates and overall reproductive success [53].

Like [17], we found that PCB was also a significant predictor of pregnancy rates, when entered with the more important variables Noise or MEDD in models with or without a term for COD. Since the explanatory variable for PCB is based on adult males, its relevance for pregnancy rates is, however, not straight forward. Data presented by [46] for the Southern North Sea show that the percentage of adult males exceeding the PCB threshold concentration suspected to cause reproductive failure in females (11mg/kg lipid weight) is 76.9% while it is 10.5% for females. This difference could be due to female offloading of PCB to calves, but still suggests a much more modest potential impact on reproductive rates beyond the first birth than estimates based on males. In juveniles, the reported percentages above the threshold of 11mg/kg lipid weight were 25% for females and 40.5% for males [46], suggesting that offloading is not the only driver of sex differences in porpoise PCB loads. It should also be noted that the estimates of PCB levels used in [17] and the present study are in many cases not synoptic with the life history data. For Norwegian porpoises, information on PCB levels is primarily based on samples collected1988-1990 [108–110], when PCB levels in the marine environment are widely found to have been significantly higher [111]. We therefore consider the modelled results for PCB inconclusive but acknowledge that PCB levels in parts of the North Sea area and the Baltic may still be high enough to impact reproductive rates through reduced general health [e.g. 23, 44, 111].

In our view, the main result of this meta-analysis is the clear identification of COD as a highly significant determinant of both the actual predicted pregnancy rates and the estimated significance of extrinsic predictors. Several previous studies have found nominally higher pregnancy rates for trauma-killed porpoises than for other COD categories [17, 18, 22], but none have so far clearly shown the significance of including COD category in models estimating the effect of extrinsic factors on harbour porpoise pregnancy rates. Similar effects of COD may be expected for other life history parameters like age at maturity and size-at-age, which are also affected by the many other sources of methodological heterogeneity outlined in the present study. In view of the increasing demands for risk assessments involving harbour porpoises, these results highlight the need for increased efforts to improve comparability between studies of harbour porpoise life history parameters.

## Supporting information

S1 Table Overview of sample units (Su) and associated reference studies for life history parameters. (DOCX)

S2 Table Details of harbour porpoise input data to reruns of meta-analyses of effects of cause of death (COD) category and extrinsic pressures on pregnancy rates. (DOCX)

S3 Table Details of predictor variables used in reruns of meta-analyses of factors affecting harbour porpoise pregnancy rates. (DOCX)

S1 Fig Tooth sections images for two harbour porpoises representing maximum and minimum deviations in age readings between R1_GLG_ and R2_Stdg_ age reading practices. (DOCX)
