# Supplementary material for: Exploring the effects of methodological choices on the estimation and biological interpretation of life history parameters for harbour porpoises in Norway and beyond
Source: PLoS One. 2024 Jul 5;19(7):e0301427. doi: 10.1371/journal.pone.0301427 (PMC11226007; doi:10.1371/journal.pone.0301427)
Supplement: S1 Table — (DOCX) [file pone.0301427.s001.docx]

**Supplementary Table S1**

Overview of sample units (Su) shown in Fig1 and associated reference studies for life history parameters listed in Tables 4 and 6. Numbers in square brackets are the reference numbers in the main text,

| **Su** | **Area** | **Years** | **[Reference nr] Reference study** |
| --- | --- | --- | --- |
| **1a** | Norway | 2016 | This study |
| **1b** | Norway | 2017 | This Study |
|  |  |  |  |
| **1c** | Norway | 1988-90 | [36] Kårstad SE. Growth and reproduction of porpoises in Norwegian and Swedish waters. MSc thesis: University of Oslo. 1993. In Norwegian |
| **2a** | Iceland | 1991-97 | [41] Ólafsdóttir D, Víkingsson GA, Halldórsson SD, Sigurjónsson J. Growth and reproduction in harbour porpoises (*Phocoena phocoena*) in Icelandic waters. NAMMCO Sci Publ. 2003; 5: 195-210. |
|  |  |  |  |
| **3a** | Scotland | 1990-‘05 | [18] Learmonth J A, Murph, S, Luque PL, Reid RJ, Patterson I AP, Brownlow A, et al. Life history of harbor porpoises (*Phocoena phocoena*) in Scottish (UK) waters. Mar Mammal Sci. 2014; 30: 1427–1455. https: doi: 10.1111/mms.12130 |
| **3b** | Western Sweden | 1988-90 | [36] Kårstad SE. Growth and reproduction of porpoises in Norwegian and Swedish waters. MSc thesis: University of Oslo. 1993. In Norwegian |
| **3c** | Denmark | 1985-91 | [59] Sørensen TB, Kinze CC. Reproduction and reproductive seasonality in Danish harbour porpoises, Phocoena phocoena. Ophelia 1994; 39(3): 159-176. |
| **3d** | Denmark | 1962-98 | [60] Lockyer C, Kinze C. Status, ecology and life history of harbour porpoise (*Phocoena phocoena*), in Danish waters. NAMMCO Sci Publ 2003; 5: 143-175. |
| **3e** | German  North Sea | 1987-‘16 | [61] Kesselring T, Viquerat S, Brehm R, Siebert U. Coming of age: - Do female harbour porpoises (*Phocoena phocoena*) from the North Sea and Baltic Sea have sufficient time to reproduce in a human influenced environment? PLoS ONE 2017; 12(10): e0186951. <https://doi.org/10.1371/journal.pone.0186951>. PMID: 29053754 |
| **3f** | Dutch  North Sea | 2006-19 | [17] Ijsseldijk LL,Hessing S, Mairo A, Doeschate MTI,Treep J, van den Broek J et al. Nutritional status and prey energy density govern reproductive success in a small cetacean. Scientific Reports 2021; 11:19201. <https://doi.org/10.1038/s41598-021-98629-x>. PMID: 34725464 |
| **3g** | South-  eastern UK | 1990-99 | [19] Murphy S, Petitguyot MAC, Jepson PD, Deaville R, Lockyer C, Barnett J et al. Spatio-Temporal Variability of Harbor Porpoise Life History Parameters in the North-East Atlantic. Front Mar Sci. 2020; 7:502352. <https://doi:10.3389/fmars.2020.502352> |
| **3h** | South-  eastern UK | 2000-12 | [19] Murphy S, Petitguyot MAC, Jepson PD, Deaville R, Lockyer C, Barnett J et al. Spatio-Temporal Variability of Harbor Porpoise Life History Parameters in the North-East Atlantic. Front Mar Sci. 2020; 7:502352. <https://doi:10.3389/fmars.2020.502352> |
| **4a** | German  Baltic Sea | 1987-‘16 | [61] Kesselring T, Viquerat S, Brehm R, Siebert U. Coming of age: - Do female harbour porpoises (*Phocoena phocoena*) from the North Sea and Baltic Sea have sufficient time to reproduce in a human influenced environment? PLoS ONE 2017; 12(10): e0186951. <https://doi.org/10.1371/journal.pone.0186951>. PMID: 29053754 |
| **5a** | Southwestern UK | 1990-99 | [19] Murphy S, Petitguyot MAC, Jepson PD, Deaville R, Lockyer C, Barnett J et al. Spatio-Temporal Variability of Harbor Porpoise Life History Parameters in the North-East Atlantic. Front Mar Sci. 2020; 7:502352. <https://doi:10.3389/fmars.2020.502352> |
| **5b** | Southwestern UK | 2000-12 | [19] Murphy S, Petitguyot MAC, Jepson PD, Deaville R, Lockyer C, Barnett J et al. Spatio-Temporal Variability of Harbor Porpoise Life History Parameters in the North-East Atlantic. Front Mar Sci. 2020; 7:502352. <https://doi:10.3389/fmars.2020.502352> |
| **6a** | Bay of Biscay | 1990-10 | [62] Read FL Santos MB, González AF, López A, Ferreira M, Vingada J. et al. 2013. Understanding harbour porpoise (*Phocoena phocoena*) and fisheries interactions in the north-west Iberian Peninsula. Final Rep. to ASCOBANS (2010). 20th ASCOBANS Advisory Committee Meeting AC20/Doc.6.1.b Warsaw, Poland, 27-29 August 2013. https://www.ascobans.org/sites/default/files/document/AC20_6.1.b_ProjectReport_PorpoiseFisheryInteractionsIberia_2.pdf |
| **7a** | West Greenland | 1988-95 | [52] Lockyer C, Heide-Jørgensen MP, Jensen J, Kinze CC, Buus Sørensen T. Age, length and reproductive parameters of harbour porpoises *Phocoena phocoena* (L.) from West Greenland. ICES J Mar Sci. 2001; 58: 154–162. |
| **8a** | Eastern  Newfoundland | 1990-91 | [37] Richardson SF. Growth and reproduction of the harbour porpoise, Phocoena phocoena, from the Eastern Newfoundland. M.Sc. Thesis, Memorial University of Newfoundland, Canada 1992: 102pp. |
| **9a** | Bay of  Fundy | 1969-73 | [63] Read, AJ, Gaskin DE. Changes in growth and reproduction of harbour porpoises, *Phocoena phocoena*, from the Bay of Fundy. Can J Fish Aquat Sci. 1990; 4721: 58-2163. |
| **9b** | Bay of  Fundy | 1985-89 | [63] Read, AJ, Gaskin DE. Changes in growth and reproduction of harbour porpoises, Phocoena phocoena, from the Bay of Fundy. Can J Fish Aquat Sci. 1990; 4721: 58-2163. Subset, early gestation pregnancy rate: [100] Read AJ. Age at sexual maturity and pregnancy rates of harbour porpoises Phocoena phocoena from the Bay of Fundy. Can J Fish Aquat Sci. 1990; 47(3): 561-565. |
| **9c** | Gulf of Maine | 1989-93 | [47] Read AJ, Hohn AA. Life in the fast lane: the life history of harbor porpoises from the Gulf of Maine. Mar Mammal Sci. 1995; 11(4): 423-440. |
| **9d** | Massa-  chussets | 1975-89 | [64] Polacheck T, Wenzel FW, Early G. What do stranding data say about harbor porpoises (Phocoena phocoena).SC/42/SM39. Rep Int Whal Commn. 1995; 16: 169-179 |
