# Supplementary material for: Exploring the effects of methodological choices on the estimation and biological interpretation of life history parameters for harbour porpoises in Norway and beyond
Source: PLoS One. 2024 Jul 5;19(7):e0301427. doi: 10.1371/journal.pone.0301427 (PMC11226007; doi:10.1371/journal.pone.0301427)
Supplement: S2 Table — (DOCX) [file pone.0301427.s002.docx]

Supplementary Table S2: Details of harbour porpoise input data to our meta-analyses of effects of cause of death (COD) category and extrinsic pressures on pregnancy rates. Meta-analysis 1 refers to our first analysis including all the 15 data points used by Ijsseldijk et al. (2021)[17].Meta-analysis 2 refers to our second analysis based only on trauma killed samples including two data points that were not used by [17] (*italics*). Location names are according to Ijsseldijk et al. (2021). The reference study for each data point is shown with both first author notation and reference number according to main text (except the present study).Entries marked with * have not been independently verified based on the original study, but only seen in the supplementary files of [17].Sample unit affiliation of each data point follows Fig.1 of the main text and is shown with associated Area Code, which was used as a random variable in both meta-analyses. The response variable of both analyses was the log odds of being pregnant calculated based on the observed number of pregnant females (N_preg_) and barren females (mature non pregnant, N_barren_) for each data point. The same numbers are used to calculate the proportion pregnant (Pregnancy rate).

| Meta-Analysis 1  All COD | Meta-  Analysis 2  Only Trauma | Location  (Naming according to [15]) | [Ref.nr.] Reference Study | COD  Category | S_u_ | Area  Code | N_preg_ | N_barren_ | Pregnancy rate |
| --- | --- | --- | --- | --- | --- | --- | --- | --- | --- |
| Included | Excluded | Salish Sea | [78] Norman et al. 2018 | Mixed | 10a | 10 | 15 | 38 | 0.28 |
| Included | Excluded | English and Welsh waters | [19] Murphy et al. 2020 | Mixed | 3g, h | 3 | 12 | 30 | 0.29 |
| Included | Excluded | Dutch waters-new | [17] Ijsseldijk et al. 2021 | Mixed | 3f | 3 | 46 | 73 | 0.39 |
| Included | Excluded | Scottish waters | [17] Learmonth et al. 2014 | Mixed | 3a | 3 | 17 | 25 | 0.40 |
| Included | Excluded | Baltic Sea | [61] Kesselring et al. 2018  ([17] Ijsseldijk et al. 2021) | Mixed | 4a | 4* | 11* | 13* | 0.46* |
| Included | Excluded | NW Iberian Peninsula | [62] Read et al. 2013 | Mixed | 6a | 6 | 7 | 6 | 0.54 |
| Included | Excluded | Kattegat Skagerrak Seas | [77] Hedlund H. 2008 | Mixed | 3b | 3* | 20* | 15* | 0.57* |
| Included | Excluded | German North Sea | [61] Kesselring et al. 2018  ([17] Ijsseldijk et al. 2021) | Mixed | 3e | 3* | 28* | 21* | 0.57* |
| Included | Excluded | Celtic Irish Seas | [19] Murphy et al. 2020 | Mixed | 5a, b | 5 | 36 | 24 | 0.60 |
| Included | Included | Danish waters new | [59] Sørensen and Kinze 1994 | Trauma | 3d | 3 | 24 | 9 | 0.73 |
| Included | Included | Bay of Fundy | [53] Read 1990 | Trauma | 9a | 9 | 26 | 9 | 0.74 |
| Included | Included | Norwegian Sea | This study | Trauma | 1a+b | 1 | *22* | 3 | 0.88 |
| Included | Included | Eastern Newfoundland | [37] Richardsson 1992 | Trauma | 8a | 8 | 15 | 2 | 0.88 |
| Included | Included | Gulf of Maine | [47] Read and Hohn 1995 | Trauma | 9c | 9 | 13 | 1 | 0.93 |
| Included | Included | Iceland waters | [41] Olafsdottir et al. 2003 | Trauma | 2a | 2 | 73 | 1 | 0.99 |
| *Excluded* | *Included* | *Dutch waters-new* | *[17] Ijsseldijk et al. 2021* | *Trauma* | *3f* | *3* | *22* | *16* | *0.58* |
| *Excluded* | *Included* | *English, Welsh and Scottish waters* | *[22] Murphy et al. 2015* | *Trauma* | *3a+3g+3h* | *3* | *10* | *10* | *0.50* |
