# Supplementary material for: Exploring the effects of methodological choices on the estimation and biological interpretation of life history parameters for harbour porpoises in Norway and beyond
Source: PLoS One. 2024 Jul 5;19(7):e0301427. doi: 10.1371/journal.pone.0301427 (PMC11226007; doi:10.1371/journal.pone.0301427)
Supplement: S3 Table — (DOCX) [file pone.0301427.s003.docx]

**Supplementary Table S3**

Details of predictor variables used in meta-analyses of factors affecting harbour porpoise pregnancy rates. The first four columns are identical to supplementary Table 2. The following two columns are minimum and maximum values of vessel noise in dB re 1uPa^2^ at 100 Hz extracted from Duarte et al. (2021) [80]. The calculated mean value was used as a covariate in the mixed model meta-analysis of harbour porpoise pregnancy rates in the present study. The information in the columns for MEDD, CHI and PCB is taken directly from Ijsseldijk et al. (2021) [17]. The last column shows the factor used in the present study to separate data points associated with relatively shallow North Sea waters from other data points. Numbers in square brackets refer to the reference numbers in the main text (including references mentioned in this caption).

| Analysis 1  All COD | Analysis 2  Only Trauma | Location  (Naming according to [17]) | COD | Noise  (Min) | Noise  (Max) | Noise  (Mean) | MEDD | CHI | PCB | NS  factor |
| --- | --- | --- | --- | --- | --- | --- | --- | --- | --- | --- |
| Incl. | Excl. | Salish Sea [78] | Mixed | 95 | 100 | 97.5 | 4.15 | 1.323 | 13.66 | NotNS |
| Incl. | Excl. | English and Welsh waters [19] | Mixed | 100 | 108 | 104 | 4.82 | 2.11 | 34.64 | NS |
| Incl. | Excl. | Dutch waters-new [17] | Mixed | 100 | 108 | 104 | 4.92 | 1.718 | 24 | NS |
| Incl. | Excl. | Scottish waters [18] | Mixed | 95 | 98 | 96.5 | 4.54 | 2.636 | 14.25 | NotNS |
| Incl. | Excl. | Baltic Sea [61] | Mixed | 110 | 110 | 110 | 5.07 | 2.106 | 27.49 | NotNS |
| Incl. | Excl. | NW Iberian Peninsula [62] | Mixed | 96 | 101 | 98.5 | 5.21 | 1.555 | 50.8 | NotNS |
| Incl. | Excl. | Kattegat Skagerrak Seas [77] | Mixed | 100 | 110 | 105 | 6.69 | 2.08 | 24.25 | NS |
| Incl. | Excl. | German North Sea [61] | Mixed | 100 | 110 | 105 | 5.11 | 1.916 | 2.2 | NS |
| Incl. | Excl. | Celtic Irish Seas [19] | Mixed | 102 | 107 | 104.5 | 6.79 | 2.106 | 43.3 | NotNS |
| Incl. | Incl. | Danish waters new [59] | Trauma | 100 | 105 | 102.5 | 4.86 | 1.987 | 29.75 | NS |
| Incl. | Incl. | Bay of Fundy [53] | Trauma | 90 | 97 | 93.5 | 6.82 | 1.208 | 82.79 | NotNS |
| Incl. | Incl. | Norwegian Sea [Present study] | Trauma | 93 | 98 | 95.5 | 5.15 | 1.942 | 21.31 | NotNS |
| Incl. | Incl. | Eastern Newfoundland [37] | Trauma | 90 | 95 | 92.5 | 7.22 | 1.833 | 11.14 | NotNS |
| Incl. | Incl. | Gulf of Maine [47] | Trauma | 90 | 97 | 93.5 | 6.12 | 1.168 | 26.8 | NotNS |
| Incl. | Incl. | Icelandic waters [41] | Trauma | 90 | 96 | 93 | 6.7 | 2.29 | 3.03 | NotNS |
| *Excl.* | *Incl.* | *Dutch waters-new [17]* | Trauma | 100 | 108 | 104 | 4.92 | 1.718 | 24 | NS |
| *Excl.* | *Incl.* | *English Welsh Scottish waters [22]* | Trauma | 100 | 108 | 104 | 4.82 | 2.1 | 34.64 | NS |
