# Supplementary material for: Exploring the effects of methodological choices on the estimation and biological interpretation of life history parameters for harbour porpoises in Norway and beyond
Source: PLoS One. 2024 Jul 5;19(7):e0301427. doi: 10.1371/journal.pone.0301427 (PMC11226007; doi:10.1371/journal.pone.0301427)

**Fig S1. (A)** Porpoise cut tooth section of female #43_2017, estimated at four years by R1_GLG_  and 22 years by R2_Stdg_. **(B)** Close-up of cementum layer of female #43_2017. **(C)** Porpoise cut tooth section of female # 44_2017 estimated at four years by both R1_GLG_ and R2_Stdg_. Note the difference in relative thickness of total cementum and dentine layer between photos A and B. Female #43_2017 was 173 cm long, pregnant and had a total of 6 CAs (5 interior and 1 surface-only CA). Female # 44_2017 was 161 cm long, pregnant and had a total of 1 CA (interior). **(C)** Close-up of cementum layer of cementum layers in female #43_2017.


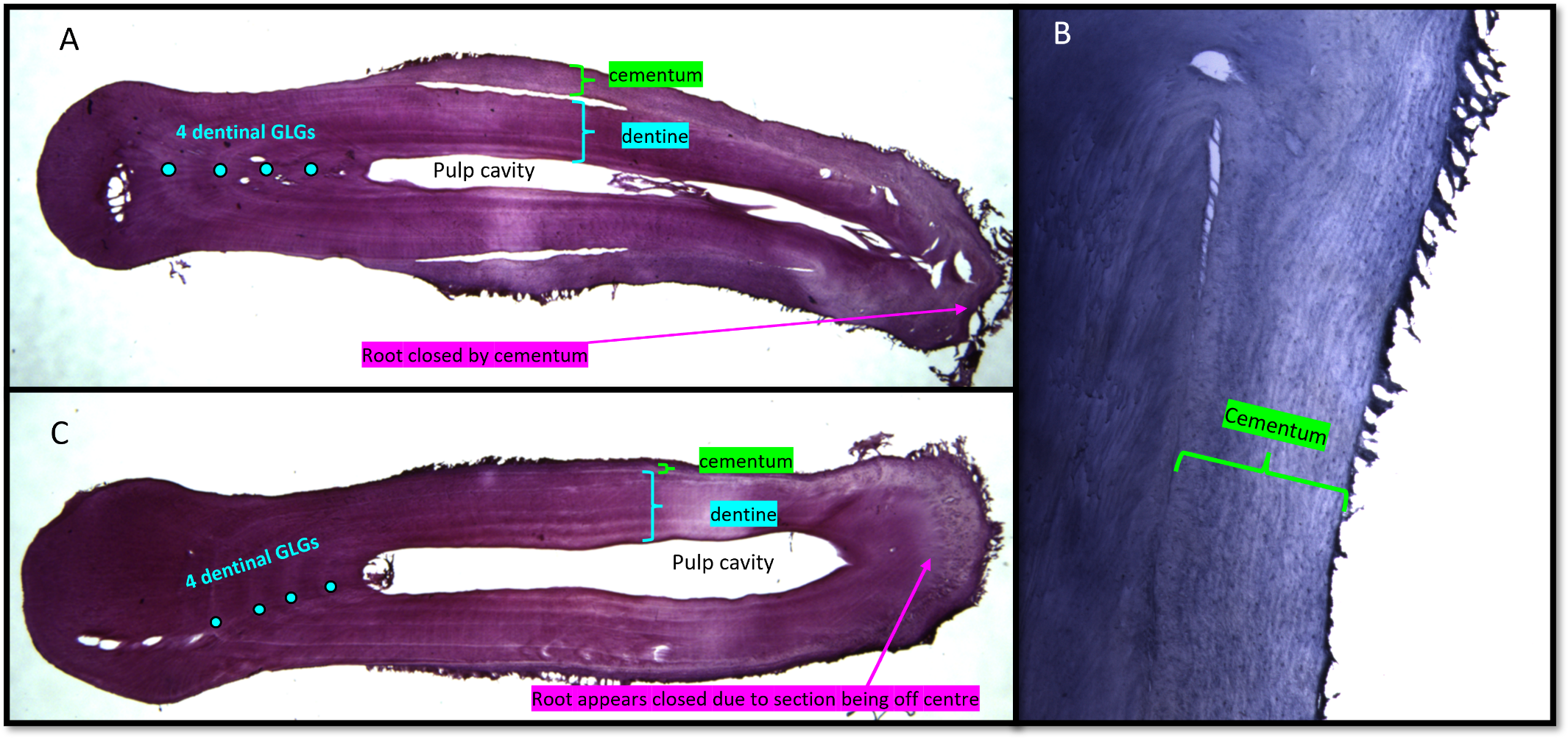


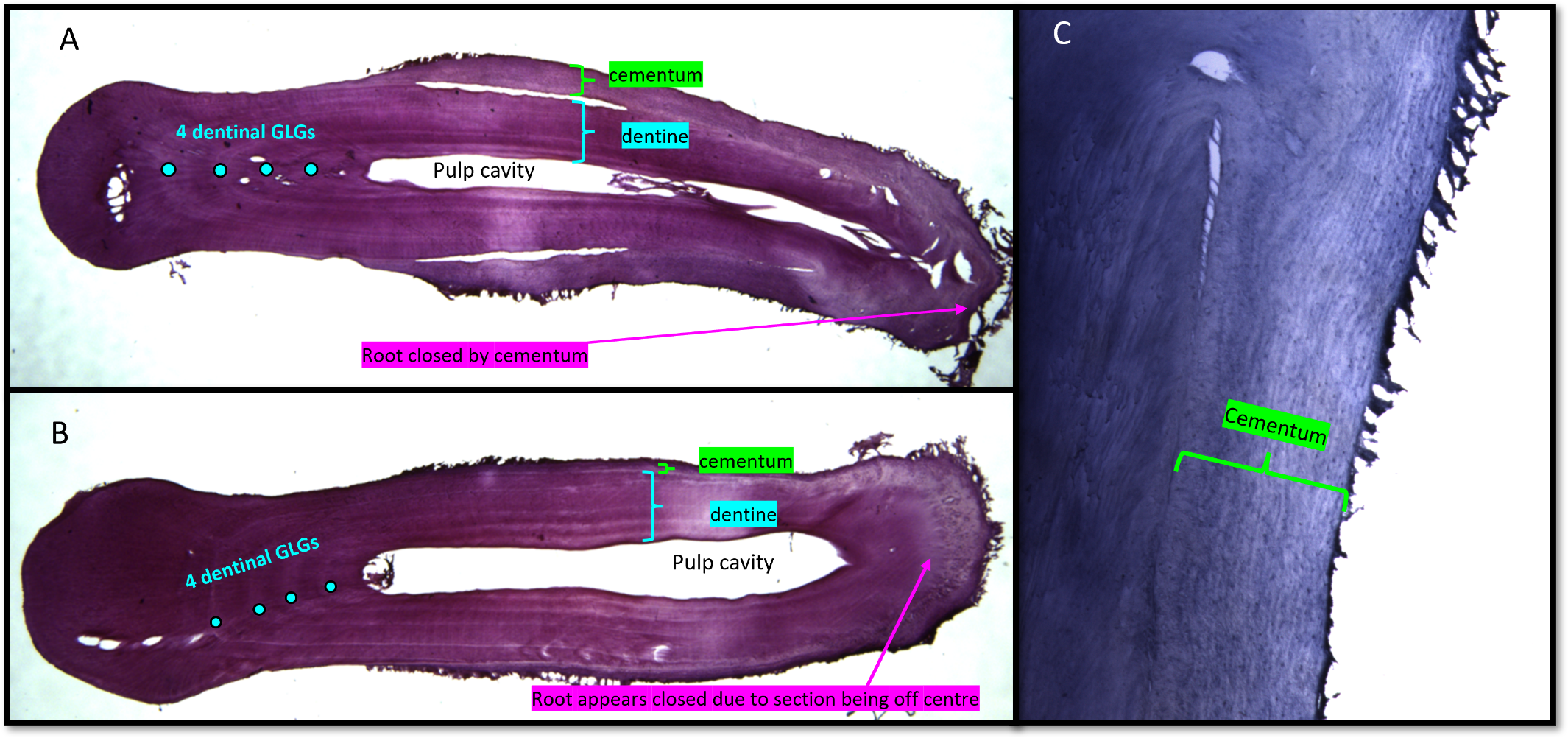

Supplement: S1 Fig — (DOCX) [file pone.0301427.s004.docx]
